# Pluripotent stem cell-derived epithelium misidentified as brain microvascular endothelium requires ETS factors to acquire vascular fate

**DOI:** 10.1073/pnas.2016950118

**Published:** 2021-02-04

**Authors:** Tyler M. Lu, Sean Houghton, Tarig Magdeldin, José Gabriel Barcia Durán, Andrew P. Minotti, Amanda Snead, Andrew Sproul, Duc-Huy T. Nguyen, Jenny Xiang, Howard A. Fine, Zev Rosenwaks, Lorenz Studer, Shahin Rafii, Dritan Agalliu, David Redmond, Raphaël Lis

**Affiliations:** ^a^Ansary Stem Cell Institute, Division of Regenerative Medicine, Department of Medicine, Weill Cornell Medicine, New York, NY 10065;; ^b^Ronald O. Perelman and Claudia Cohen Center for Reproductive Medicine, Weill Cornell Medicine, New York, NY 10065;; ^c^Department of Neurology and the Sandra and Edward Meyer Cancer Center, Weill Cornell Medicine-New York Presbyterian Hospital, New York, NY 10065;; ^d^Developmental Biology, the Center for Stem Cell Biology, Memorial Sloan Kettering Cancer Center, New York, NY 10065;; ^e^The Biochemistry, Structural Biology, Cell Biology, Developmental Biology and Molecular Biology Allied Program, Weill Cornell Graduate School of Medical Sciences, New York, NY 10065;; ^f^Department of Pathology and Cell Biology, Columbia University Irving Medical Center, New York, NY 10032;; ^g^Taub Institute for Research on Alzheimer’s Disease and the Aging Brain, Columbia University Irving Medical Center, New York, NY 10032;; ^h^Genomics Resources Core Facility, Weill Cornell Medicine, New York, NY 10065;; ^i^Department of Neurology, Columbia University Irving Medical Center, New York, NY 10032

**Keywords:** endothelial cells, induced pluripotent, single-cell RNA sequencing, blood–brain barrier, cellular identity

## Abstract

Cells derived from pluripotent sources in vitro must resemble those found in vivo as closely as possible at both transcriptional and functional levels in order to be a useful tool for studying diseases and developing therapeutics. Recently, differentiation of human pluripotent stem cells (hPSCs) into brain microvascular endothelial cells (ECs) with blood–brain barrier (BBB)-like properties has been reported. These cells have since been used as a robust in vitro BBB model for drug delivery and mechanistic understanding of neurological diseases. However, the precise cellular identity of these induced brain microvascular endothelial cells (iBMECs) has not been well described. Employing a comprehensive transcriptomic metaanalysis of previously published hPSC-derived cells validated by physiological assays, we demonstrate that iBMECs lack functional attributes of ECs since they are deficient in vascular lineage genes while expressing clusters of genes related to the neuroectodermal epithelial lineage (Epi-iBMEC). Overexpression of key endothelial ETS transcription factors (*ETV2*, *ERG*, and *FLI1*) reprograms Epi-iBMECs into authentic endothelial cells that are congruent with bona fide endothelium at both transcriptomic as well as some functional levels. This approach could eventually be used to develop a robust human BBB model in vitro that resembles the human brain EC in vivo for functional studies and drug discovery.

## Significance

Human PSC-derived iBMECs have been generated to study disease mechanisms and drug development for neurological disorders. However, their full transcriptomic characterization is unclear, which could result in inaccurate physiological studies and development of treatments with ineffective clinical outcomes. Utilizing a comprehensive transcriptomic metaanalysis validated by physiological studies, we find that many current protocols used to generate iBMECs produce a homogenous epithelial cell population. Overexpression of ETS transcription factors reprogram these cells into phenotypic endothelial cells (rECs) which recapitulate certain vascular functions, albeit lacking expression of some organotypic transporter genes and high electrical resistance in vitro. Nevertheless, they represent a crucial step toward the generation of an in vitro**model suitable for physiological and pharmaceutical studies of the blood–brain barrier.

Over the past decade, multiple stem cell biology laboratories have aimed to generate various cell types in vitro either by coaxing pluripotent stem cells (PSCs) to differentiate along developmental lineages into target cell types or by converting one somatic cell type to another with exogenous expression of specific transcription factors (TFs). Initial studies describing how a given cell fate, comparable to native cells, could be achieved by directed differentiation of PSCs were established based on the analysis of a small number of cell surface markers, restricted functional assays, and a targeted survey of gene expression profiles that provided, at best, a qualitative suggestion of similarity to the native cell. However, these approaches lack an unbiased metric of cellular identity.

**Fig. 1.**
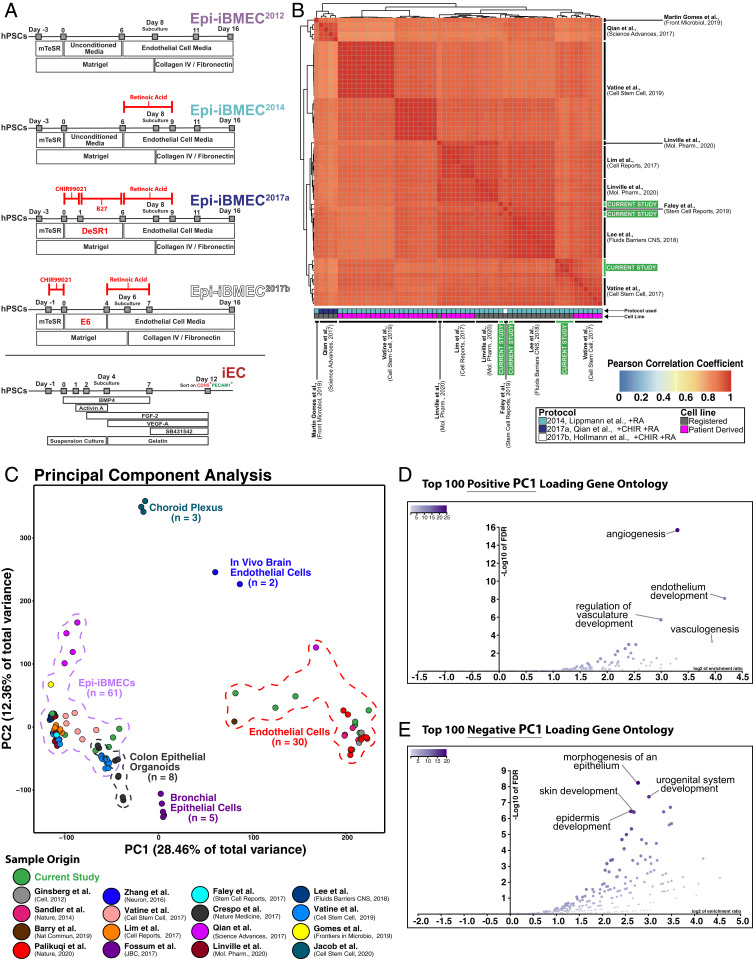
Metaanalysis of global high-throughput gene expression profiles reveal Epi-iBMECs possess an epithelial transcriptomic signature. (*A*) Schematic diagrams for differentiation of hPSCs to Epi-iBMECs highlighting changes (marked in red) implemented since its initial description as well as differentiation of hPSCs into generic endothelial cells (iECs). (*B*) Heatmap showing Pearson correlation coefficients between previously reported Epi-iBMEC transcriptomes and Epi-iBMECs generated in the current study. Epi-iBMECs generated by our group are molecularly equivalent to those reported in the literature. (*C*) Principal component analysis plot approximating relative relationship of 109 distinct cell samples across 22 library preparations from previously published and newly generated bulk RNA-sequencing data. (*D*) Volcano plots depicting gene ontology of biological processes using the top 100 positive loading genes of PC1 demonstrating expression of genes involved in epithelial cell processes. (*E*) Volcano plots depicting gene ontology of biological processes using the top 100 negative loading genes of PC1, demonstrating expression of genes involved in endothelial cell processes.

Here, we assess the cellular identity of human pluripotent stem cell (hPSC)-derived brain microvascular endothelial cells (iBMECs) possessing blood–brain barrier (BBB) attributes developed by Lippmann et al. (1) which has been both used and modified in various subsequent studies (2–6, 9, 11, 13–24) (Fig. 1*A*). These cells have been reported to meet the need for a reliable and reproducible in vitro model of the human BBB that can be used to screen drugs and understand mechanisms of neurological diseases (25, 26). This benchmark protocol relies on performing a sequence of defined steps starting with the differentiation of PSCs into both neural and endothelial lineages. A human serum-free endothelial base medium is then used to stimulate EC expansion and finally, the heterogenous population of cells is subcultured from Matrigel onto a CollagenIV/Fibronectin matrix. This addition of supportive extracellular physical cues is meant to bolster endothelial cell (EC) maturation and result in a pure population of iBMECs.

Since the initial report (1), the authors sought to improve the quality and the yield of these iBMECs resulting in three major iterations of the original protocol. In 2014, it was demonstrated that addition of 10 µM retinoic acid (RA) during expansion [day 6 in endothelial cell medium (ECM)] induces CDH5 (VE-Cadherin) expression prior to subculturing and enhances BBB properties (e.g., expression of tight junction proteins and transendothelial electrical resistance [TEER]) (2). In 2017, iBMECs were generated by sequential activation of Wnt/beta-catenin and retinoic acid signaling (9). Compared to the initial differentiation method reported in Lippmann et al. (1) the cells generated under sequential activation of Wnt and RA exhibited higher TEERs and lower batch-to-batch variations (9). Lastly, also in 2017, it was reported that the use of a defined medium (E8) accelerates the differentiation of hPSCs to iBMECs while achieving comparable properties to iBMECs produced by prior methods (18). Notwithstanding these refinements, all methods claim to promote differentiation of hPSCs into brain microvascular ECs that are phenotypically (GLUT1^+^PECAM1^+^CDH5^+^) endothelium and display some endothelial-like properties such as low-density lipoprotein uptake, high TEER, and barrier-like efflux transporter activities (1, 2, 4–12). In summary, iBMECs can be obtained from PSCs using either the original method (1) or subsequently derived protocols (1, 2, 9, 18). However, it is unclear how similar these cells are to BBB-forming ECs found in the central nervous system (CNS) or even generic ECs differentiated from the same hPSC lines (iECs) using a previously established protocol (27).

Using a combination of bulk and single-cell RNA sequencing approaches combined with metaanalysis of our and previously published transcriptomic data as well as immunofluorescence, we set out to characterize how closely the cellular identity of iBMECs generated by these differentiation protocols resembles bona fide ECs. In contrast to the published reports, we find that iBMECs obtained by these differentiation protocols lack canonical endothelial cell markers (CDH5, PECAM1, KDR (VEGFR2), APLNR, and eNOS) as well as some critical ETS transcription factors ETS1, ETV6, and FLI1, among others which are crucial for establishing a vascular endothelial identity. This lack of expression is true even when compared to their iEC counterparts. iBMECs do not form lumenized vessels in immunocompromised mice (NOD-scid IL2Rg^null^-SGM3) nor do they demonstrate a canonical endothelial response to inflammatory stimulus. Moreover, at a molecular level, iBMECs express high levels of EpCAM and exhibit a gene signature typical of neuroectodermal epithelial cells. Analysis by bulk and single-cell RNA sequencing reveals that expression of multiple epithelial-related genes (28, 29) such as CDH1, CDH3, CLDN4, and KRT7 is also present in EpCAM^+^ iBMECs (Epi-iBMECs).

Finally, we demonstrate that Epi-iBMECs can only be directed toward an endothelial fate by overexpression of three key endothelial ETS transcription factors *ETV2*, *FLI1*, and *ERG* (30). Thus, similar to hematopoietic stem and progenitor cells (31, 32), directed differentiation of pluripotent stem cells into human ECs requires introduction of key EC TFs which could ultimately lead to the development a robust human BBB model in vitro to be used for functional studies and drug discovery.

## Results

In order to determine the extent that technical variables influence the reproducibility of the transcriptomic signature present in Epi-iBMECs, we surveyed publicly available RNA sequencing (RNA-seq) datasets generated using either the original method (1) or subsequently derived protocols (1, 2, 9, 18) (Fig. 1*A*). We generated Epi-iBMECs as previously described (15), utilizing multiple hPSC lines while maintaining precise conditions and seeding densities for optimal differentiation according to published protocol. RNA sequencing was then conducted on these samples and the data were compiled with the existing datasets. Biological signal was distinguished from the noise related to experimental heterogeneity by clustering the previously published Epi-iBMECs samples with our own based on a Pearson correlation matrix calculated from log counts per million reads. The resulting heatmap displays agreement between sample annotation and cluster assignment based on correlated gene expression (Fig. 1*B* and Dataset S1). As shown, the transcriptome of Epi-iBMECs differentiated using the protocol described in Lippmann et al. (2) and replicated in Vatine et al. (11, 12), and Lim et al. (33), correlates with a high degree of confidence with the transcriptome of Epi-iBMECs generated using sequential activation of Wnt and RA signaling described in Qian et al. (9), or the latest accelerated procedure described in Faley et al. (6). Therefore, Epi-iBMEC differentiation can be achieved with a remarkable molecular homogeneity regardless of the protocol used to generate them (2–6, 9, 11, 13–15, 18–24).

We then set out to characterize the cellular identity of Epi-iBMECs since they have been described as brain microvascular ECs derived from hPSCs that demonstrate BBB properties. We compared 109 samples from 22 libraries of global gene expression profiles captured by high-throughput RNA sequencing. These included 61 Epi-iBMEC samples from 10 libraries, endothelial (comprising adult, fetal, and hPSC-derived cells) and epithelial cell (bronchial and hPSC-derived colon) controls as well as three choroid plexus organoid samples (Fig. 1*C* and *SI Appendix*, Fig. S1*A*). We also used a previously established protocol (27) to generate generic hPSC-derived ECs (iECs) from the same starting hPSC lines to test for any intrinsic bias in our starting cells (Fig. 1*A*). To compare such a diverse set of libraries that likely contain sequencing bias based on the method of RNA preparation, we analyzed and functionally annotated the top principal components (PCs). The preponderance of the molecular distances across all samples is captured within PC1 (28.5%), PC2 (12.4%), and PC3 (10.3%) (PC1+PC2+PC3 = 51.2%) (*SI Appendix*, Fig. S1*B*). Functional annotation of the top 100 positive loading genes by PC1 yielded biological processes associated with “angiogenesis,” “endothelial development,” “vasculogenesis,” and “regulation of vasculature development” (Fig. 1*D*). On the contrary, the top 100 negative PC1 loading genes scored biological processes such as “morphogenesis of an epithelium,” “urogenital system development,” “skin development,” and “epidermis development” (Fig. 1*E*). Based on this analysis, PC1 resolves some aspect of cellular identity (endothelial vs. epithelial) across samples (*SI Appendix*, Fig. S2*A* and Dataset S2). Similar analysis of PC2 positive and negative loading genes reveals the resolution of aspects relating to cell signaling and transporter profiles (*SI Appendix*, Fig. S2*B*). Thus, we focus on PC1 for subsequent analyses as a benchmark of cellular identity.

**Fig. 2.**
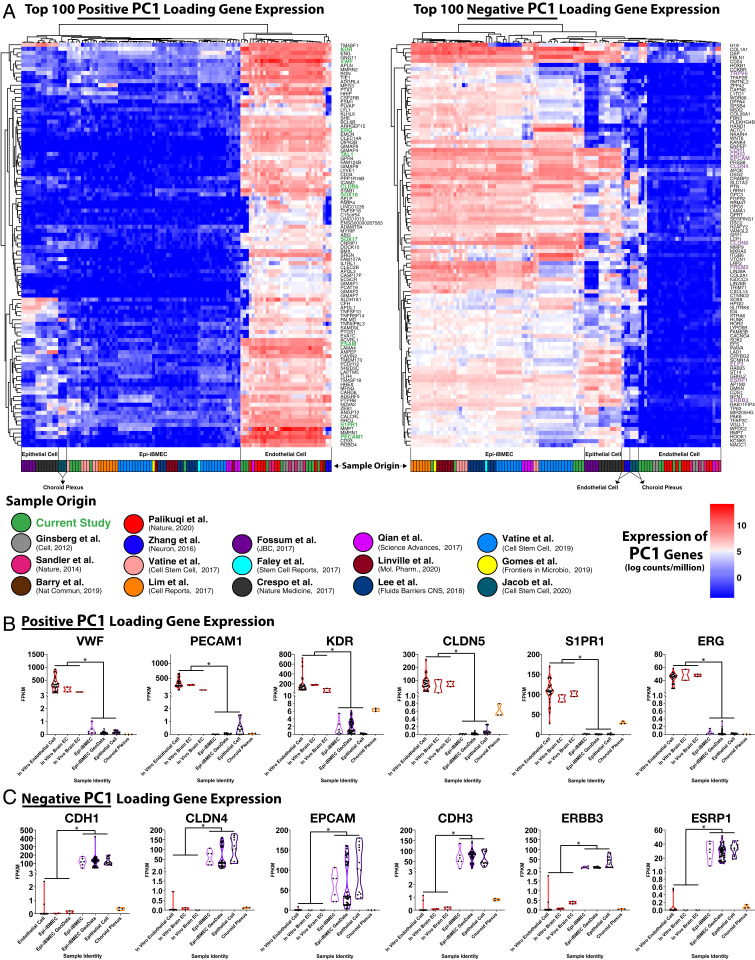
Characterization of endothelial and epithelial cell gene expression profiles in primary and hPSC-derived cells. (*A*) Heatmap illustrating expression of the top 100 most significant negative and positive loading genes of PC1 generated from unsupervised analysis of all bulk RNA gene expression profiles analyzed in Fig. 1*C*. (*B*) Violin plots of key endothelial cell genes acquired from top positive loading genes of PC1 of the bulk RNA expression profile analysis, demonstrating significant differences in expression among endothelial cell and both Epi-iBMEC as well as epithelial cell populations. (significance indicates *P value <0.05). (*C*) Violin plots of key epithelial cell genes acquired from top negative loading genes of PC1 of the bulk RNA expression profile analysis, demonstrating significant differences in expression among endothelial cell and both Epi-iBMEC as well as epithelial cell populations. (significance indicates *P value <0.05).

Clustering based upon the top 100 positive and negative PC1-loading genes demonstrated that all Epi-iBMECs cluster away from their generic iEC counterparts as well as the adult EC controls (Fig. 2*A*). This analysis revealed that Epi-iBMECs mainly expressed genes characteristic of the epithelial lineage (among them, *TRPV6*, *CDH1*, *CDH3*, *EPCAM*, *CLDN4*, *CLDN6*, *FREM2*, *ELF3*, *ESRP1*, and *ERBB3*), while lacking most of the definitive transcripts essential for the development and maintenance of an endothelial lineage/fate, including *KDR*, *VWF*, *ERGv TAL1*, *CLDN5*, *SOX18*, *SOX17*, *ESAM*, *S1PR1*, and *PECAM1* (Fig. 2 *B* and *C* and Dataset S2). In addition, our Epi-iBMECs demonstrated expression of several select tight junction proteins (ZO-1, Occludin) localized at cell–cell junctions and formed a high TEER as previously reported (1, 2, 4–12) (*SI Appendix*, Fig. S1 *C* and *D*). We conclude from this analysis that Epi-iBMECs do not have an EC transcriptome, but instead harbor unequivocal characteristics of an epithelial cell identity.

**Fig. 3.**
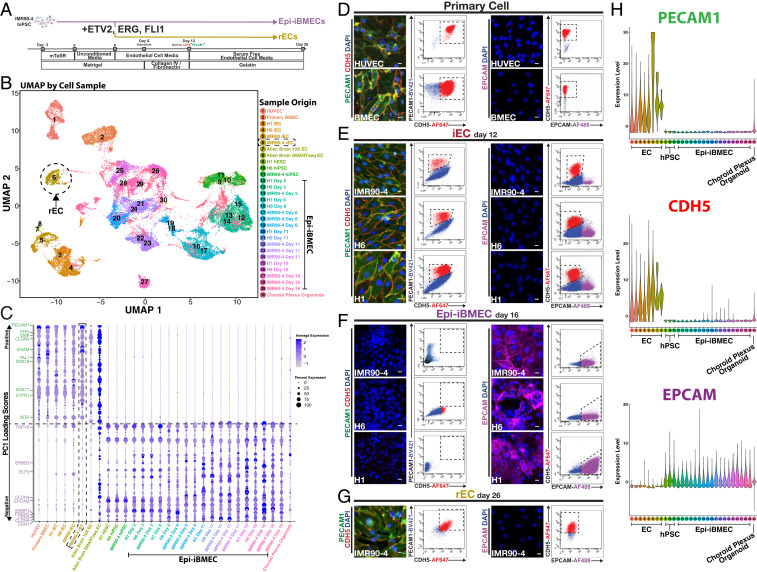
Single-cell RNA sequencing resolves transcriptomic composition of iBMECs and demonstrates rescue of a vascular identity upon transduction of three transcription factors. (*A*) Schematic diagram for the generation of rECs highlighting the induction of ETV2, ERG, and FLI1 expression at day 6 of the Epi-iBMEC differentiation as well as FACS isolation of CDH5^+^PECAM1^+^ cells at day 12 followed by expansion on 0.1% gelatin with serum-free EC media. (*B*) sc-RNA expression profiles displayed on a UMAP plot illustrate 30 distinct cell samples used in analysis of primary ECs, iECs, and Epi-iBMECs at various days of differentiation and hPSCs as well as choroid plexus cells. rECs are highlighted to illustrate that the endothelial transcriptomic signature of Epi-iBMECs is rescued upon induction of ETV2, ERG, and FLI1. (*C*) Heatmap emphasizing differences in expression levels of both endothelial- and epithelial-specific genes in all cell samples from Fig. 2*B*. Genes were derived from PC1 of unsupervised bulk RNA analysis depicted in Fig. 1*C*. The rEC sample (outlined) shows restoration of both EC-specific gene expression to levels comparable to those of EC controls. (*D*) Confocal microscopy and flow cytometry of control ECs (HUVEC and BMEC) for PECAM1 (green), CDH5 (red), EPCAM (purple), and DAPI (blue). Representative plots of *n* = 5 biological replicates. (Scale bars, 50 µm.) (*E*) Confocal microscopy and flow cytometry of control hPSC-derived iECs for PECAM1 (green), CDH5 (red), EPCAM (purple), and DAPI (blue). Representative plots of *n* = 5 biological replicates. (Scale bars, 50 µm.) (*F*) Confocal microscopy and flow cytometry of control hPSC-derived Epi-iBMECs for PECAM1 (green), CDH5 (red), EPCAM (purple), and DAPI (blue). Representative plots of *n* = 5 biological replicates. (Scale bars, 50 µm.) (*G*) Confocal microscopy and flow cytometry of rECs for PECAM1 (green), CDH5 (red), EPCAM (purple), and DAPI (blue). Representative plots of *n* = 5 biological replicates. (Scale bars, 50 µm.) (*H*) Violin plots of the samples from the sc-RNA sequencing analysis confirming expression of PECAM1 and CDH5 in all endothelial cell samples to be far higher than Epi-iBMEC samples. EPCAM is shown to be expressed at a much higher level in Epi-iBMEC samples compared to all endothelial cell samples.

We have previously shown that lineage-committed epithelial cells (EpCAM^+^Tra1-81^−^c-Kit^−^) from amniotic fluid are amenable to transcription factor-mediated reprogramming into vascular ECs (30). Overexpression of three ETS transcription factors, *ETV2*, *ERG*, and *FLI1*, not only induced a stable endothelial transcriptomic profile in committed epithelial cells, but also suppressed expression of epithelial genes (30). We therefore transduced IMR90-4 iPSC-derived Epi-iBMECs with *ETV2*, *ERG*, and *FLI1* (*EEF*) at day 6 of the neuroendothelial differentiation protocol, when endothelial fate is supposed to be established (1–3), and allowed *EEF* to be expressed for the duration of differentiation (*Methods* and Fig. 3*A*). Remarkably, analysis of bulk RNA sequencing transcriptomes shows expression levels of key EC genes and angiocrine factors (34) in *EEF*-reprogrammed Epi-iBMECs (rECs) comparable to those of iECs and adult ECs, supporting the notion that rECs possess an endothelial identity (*SI Appendix*, Fig. S3 *A*–*C* and Dataset S2).

From here we sought to further characterize the rECs and assess their endothelial function as well as their potential for use in a BBB model. The *CLDN* (Claudin) gene family encodes for a variety of proteins which are essential for tight junction formation and permeability modulation, with specific claudins native to certain tissues such as *CLDN5* which is specific to ECs (35). Furthermore, membrane transporters such as solute carriers (SLC transporters) as well as adenosine triphosphate (ATP) binding cassette (ABC) transporters are essential for supplying nutrients to the CNS through the BBB (36). As expression levels of these genes are crucial to the function and maintenance of the BBB in vivo, we analyzed the transcriptomic data for expression of various brain endothelial cell transporters as well as Claudin genes (*SI Appendix*, Figs. S4–S6). This metaanalysis revealed that expression of organotypic brain endothelial cell transporters were significantly higher in the in vivo brain EC samples. Additionally, we found no evidence that Epi-iBMECs express many brain EC-specific transporters (*SLCO1A2*, *SLCO1C1*, *MFSD2A*, *ABCG2*, and *ABCB1*) or endothelial CLDN genes (*CHD5*); however, they express an epithelial cell CLDN gene repertoire (*CLDN3*, *CLDN6*, and *CLDN7*), raising concerns for their use as an efficacious vascular BBB model. The rEC samples lacked certain brain EC-specific transporter genes and more closely resembled the generic iECs and other in vitro EC samples.

Though rECs were shown to be more of a generic EC, lacking an organotypic transcriptomic profile, they could be sorted and expanded as stable, phenotypically marked cells with EC identity (CDH5^+^PECAM1^+^EPCAM^−^) for at least 2 wk in culture under TGF-β signaling inhibition (*SI Appendix*, Fig. S7*A*). Further characterization of rECs confirmed protein expression of ZO-1 and Occludin confirming presence of tight junctions by fluorescence microscopy (*SI Appendix*, Fig. S1*E*). Additionally, when stimulated with either VEGF-A or an anti-VE-cadherin antibody (BV9), rECs showed an increase in vascular permeability to 70 kDa dextran comparable to HUVECs. In contrast, Epi-iBMECs do not show any changes in permeability with the same stimulation, validating their lack of *KDR* expression (*SI Appendix*, Fig. S1*F*). We also report decreased TEER values observed when purified rECs were compared to Epi-iBMECs starting at day 8 of their differentiation when TEER measurements have previously been reported (*SI Appendix*, Fig. S1*G*).

Directed differentiation of a target cell type from hPSCs often yields a heterogeneous population where the target cells are less frequent. Therefore, the transcriptomic signature of the target cell could potentially be lost when gene expression profiling is performed on the ensemble of cells that form a bulk population. Since the generation of Epi-iBMECs relies on a selection step (1–3), the epithelial signature of Epi-iBMECs (Fig. 2*C*) may reflect the presence of some cell contaminants. Moreover, we set out to determine whether expression of *ETV2*, *ERG*, and *FLI1* was sufficient to confer a homogenous molecular signature congruent with an EC fate in all rECs, in addition to inducing EC phenotypic protein expression. To address these issues, we sequenced the transcriptomes of 32,939 individual cells comparing our rECs to Epi-iBMECs, iECs, adult ECs, and choroid plexus organoid controls. All samples were generated and sequenced by our group with the exception of two brain EC samples and one choroid plexus sample acquired from the Allen Institute Brain Map (37, 38) and Pellegrini et al. (39), respectively. Single-cell transcriptomes were sequenced with an average depth of 9,443 unique molecular identifiers (UMI) and 2,669 genes per cell (*SI Appendix*, Fig. S7*B*). In order to detect relationships among cells from various differentiation protocols and sources, we visualized all cells by uniform manifold approximation and projection (UMAP; Fig. 3*B*). The clustering of the 30 samples shows a clear disparity between the cellular identity of Epi-iBMECs and (both iEC and adult) ECs and confirms a rescue of the endothelial transcriptome in rECs (Fig. 3*B*). We investigated how the top and bottom 100 PC1 loading genes, identified in Fig. 2*A*, contribute to cell type identity in our single-cell RNA sequencing dataset by evaluating average gene expression profiles for each cell type (Fig. 3*C*). Analysis of these data confirmed that we generated a homogeneous population of Epi-iBMECs comparable to those described in the literature (1, 2, 4–12) at the transcriptome level (Fig. 3*C*).

Our single-cell transcriptomic analysis confirmed that expression of a significant number of vascular genes curated from PC1 (*KDR*, *VWF*, *ERG*, *TAL1*, *CLDN5*, *SOX18*, *SOX17*, *ESAM*, *S1PR1*, and *PECAM1*) was absent in Epi-iBMECs, but these transcripts were reestablished in rECs. Moreover, rECs lost expression of all transcripts associated with an epithelial lineage (*TRPV6*, *CDH1*, *CDH3*, *EPCAM*, *CLDN4*, *CLDN6*, *FREM2*, *ELF3*, *ESRP1*, and *ERBB3*) that were present in Epi-iBMECs (Fig. 3*C* and *SI Appendix*, Fig. S3 *C* and *D*). A principal component analysis of this single-cell data shows a clear divergence between all our EC samples from the Epi-iBMEC and choroid plexus organoid samples across PC1 (*SI Appendix*, Fig. S8*A*). We performed a gene set enrichment analysis of biological processes on all of the positive and negative loading genes contributing to this new single-cell PC1 (*SI Appendix*, Fig. S8*B*) and found almost an 80% intersection between the single-cell and bulk RNA PC1 genes (*SI Appendix*, Fig. S8*C*). Taken together, this analysis substantiated our initial findings from bulk RNA sequencing that the divergence between Epi-iBMECs and all EC controls across PC1 reflects an intrinsic difference in cell identity, where all individual Epi-iBMECs are annotated as epithelial rather than endothelial cells. This combined dataset also shows that we cannot capture any one cell, generated with the Epi-iBMEC differentiation protocols described in previous literature (1–3) (Fig. 1*A*) that contains an EC molecular signature comparable to in vivo brain ECs as defined in recently published studies (40) as well as the Allen Institute Brain Map reference data (*SI Appendix*, Fig. S9). Therefore, if the Epi-iBMECs derivatives from these protocols can yield ECs (with or without BBB properties), the observation rate of these events would appear to be very low (fewer than 1 in 18,043 iBMECs sequenced in this study). However, introduction of *EEF* is sufficient to establish a nonbrain-specific EC molecular signature in Epi-iBMECs.

We validated the identity of rECs by comparing expression of a variety of endothelial and epithelial markers in stable rECs (day 26) with Epi-iBMECs and iECs derived from either induced PSCs (IMR90-4 and H6) or ESCs (H1), human umbilical vein endothelial cells (HUVECs) and BMECs (positive EC controls) by confocal microscopy and fluorescence-activated cell sorting (FACS) (Fig. 3 *D*–*G*). Epi-iBMECs were assayed at day 16 as this is the stage of differentiation when their TEER stabilizes in vitro (*SI Appendix*, Fig. S1*D*). We found that Epi-iBMECs lack expression of canonical EC markers (CDH5 and PECAM1) by day 16 as opposed to rECs, iECs, or adult EC controls (HUVECs and BMECs; Fig. 3 *D*–*G*) in agreement with the transcript expression capture by single-cell RNA sequencing (Fig. 3*H*). In contrast, Epi-iBMECs, regardless of the cell line of origin, were the only cells that express canonical epithelial marker EPCAM by both confocal microscopy and FACS. However, *EPCAM* transcript and protein expression were silenced in rECs by day 12, confirming loss of epithelial identity in iBMECs upon expression of *EEF* (Fig. 3 *D*–*H*).

**Fig. 4.**
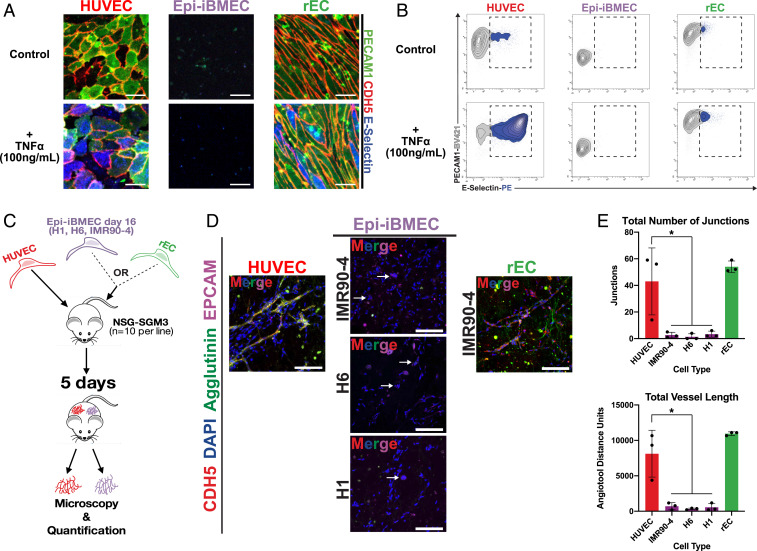
Transduction of ETV2, ERG, and FLI1 promotes functional properties characteristic of an endothelial cell in Epi-iBMECs. (*A*) Confocal images for E-Selectin (blue), PECAM1 (green), and CDH5 (red) illustrate an increase in E-Selectin (CD62E) protein expression at the cell membrane upon addition of 100 ng mL^−1^ tumor necrosis factor alpha (TNFα) in HUVEC and rEC samples but not in Epi-iBMEC samples. *n* = 5 biological replicates (Scale bars, 50 µm.) (*B*) Flow cytometry plots for E-Selectin and PECAM1 confirm increased E-Selectin protein expression in HUVEC and rEC samples upon stimulation with 100 ng mL^−1^ TNFα in contrast to Epi-iBMECs. (*C*) Schematic illustration of the in vivo tubulogenesis assay for the angiogenic potential of Epi-iBMECs (derived from H1, H6, and IMR90-4 at day 16 of differentiation), rECs and HUVECs in Matrigel plugs. Cells were mixed into Matrigel and s.c. injected into NSG-SGM3 mice. Plugs were excised 5 d postinjection, cryosectioned, and analyzed for vessel formation. (*D*) Confocal microscopy images for DAPI (blue), EpCAM (purple), Agglutinin (green), and CDH5 (red) depicting in vivo formation of vessel-like structures in HUVEC and rEC plugs. White arrows in Epi-iBMEC panels show groups of cells forming EpCAM^+^ cell clusters. *n* = 10 mice per experimental cell line. (*E*) Quantifications of in vivo tubulogenesis assay with the AngioTool. The graph on the *Top* shows the total number of vessel junctions in each sample, whereas the one on the *Bottom* shows the total length of all vessels in each sample. *n* = 3 biological replicates, *n* = 10 mice per cell line tested. (significance indicates *P value <0.05).

Subsequently, we tested whether overexpression of *EEF* is also sufficient to confer Epi-iBMECs the ability to respond to proinflammatory cues in vitro and exhibit an angiogenic potential to drive formation of a capillary network in vivo. E-selectin (CD62E) is a cell adhesion molecule expressed by both tissue-specific and generic EC upon activation by proinflammatory signals (41, 42). We stimulated rECs for 3 h with 100 ng mL^−1^ tumor necrosis factor alpha (TNFα) and examined their ability to induce expression and mobilize E-selectin to the plasma membrane by confocal microscopy and flow cytometry. rECs responded to TNFα by up-regulating and mobilizing E-selectin to the plasma membrane, at a lower level than detected in the EC controls, in contrast to control Epi-iBMECs, which did not up-regulate E-selectin upon TNFα exposure (Fig. 4 *A* and *B*). Thus, rECs show some ability to integrate proinflammatory signals in a canonical EC-fate manner while also maintaining their EC phenotype under an inflammatory stress.

Finally, we assessed the ability of rECs to form capillary or blood vessels when injected subcutaneously (s.c.) into adult, immunocompromised NSG-SGM3 mice using the Matrigel plug assay (*Methods* and Fig. 4*C*). On day 5 postinjection, plugs were excised and assayed for vessel formation by immunostaining against human CDH5 (VE-cadherin), Agglutinin, and EPCAM (Fig. 4*D*). rECs were able to form a vascular network in vivo comparable to that formed by HUVEC cells, in contrast to untransduced Epi-iBMECs that could not form vessels in vivo. Results from this assay were quantified using AngioTool following the methods described in the original publication (43) (Fig. 4*E*). Overall, the results acquired from these functional assays and fluorescence microscopy bolstered by the analysis of a combination of bulk and single-cell sequencing data allows for the conclusion that Epi-iBMECs do not possess any semblance of a vascular endothelial identity. These data also collectively demonstrate that ectopic expression of *EEF* induces and stabilizes the endothelial fate of rECs rendering them capable of responding appropriately to both proinflammatory and angiogenic stimuli.

## Discussion

Over the past decade many groups aimed to advance in vitro models of the BBB to circumvent the technical complexity of studying the BBB in vivo. The main difficulty of designing such models is to phenocopy the high TEER observed in vivo considering that BBB traits are not intrinsic to ECs, but rather the result of complex interactions with other cell types such as pericytes and astrocytes (44). Consequently, brain microvascular ECs lose their BBB properties, especially high TEER, when cultured in vitro (45–48). To resolve this issue many laboratories have developed various in vitro BBB models through the use of cocultures with pericytes and astrocytes (49), induced pluripotent stem cell differentiation (1, 2, 7, 18), brain organoids (50), and “organ-on-a-chip” approaches (14). These in vitro models are generally validated by measuring TEER as a readout of tight junction function and expression of a restricted set of BBB-EC markers. Using TEER measurement as a proxy for functional BBB tight junctions has some limitations, since it can only measure the paracellular, as opposed to transcellular, permeability. Moreover, nonendothelial cell types including epithelial cells can also display a high TEER (51). Problems may also arise from assigning EC identity based on the expression of a restricted set of brain ECs as it can often result in false positive results due to antibody cross-reactivity with proteins present in other nonendothelial cell types (52, 53).

In this study, we have used high-throughput transcriptomics to evaluate the cellular identity of Epi-iBMECs that have been extensively used for in vitro models of the human BBB. Through a rigorous metaanalysis of transcriptomic data from previously published studies combined with samples generated by our group, we substantiate a high degree of correlation between previously published Epi-iBMECs and those generated by our group. Our metaanalysis also demonstrates that all Epi-iBMECs are transcriptionally analogous to epithelial cells and have no semblance of an endothelial cellular identity as assigned by an unbiased analysis of bulk RNA sequencing data. Additionally, iECs derived from the same hPSC starting populations are EPCAM^−^PECAM1^+^CDH5^+^ cells and express canonical EC genes. Therefore, there is no inherent restriction of human PSCs to acquire EC properties. The same metaanalysis also shows a primary BMEC control from one of the previously published Epi-iBMEC datasets closely clusters with our primary EC controls, allowing us to conclude that sequencing bias and batch effect do not account for any major differences in the assigned cell identities.

Comparison of 18,863 single Epi-iBMEC transcriptomes against endothelial cells isolated from human brain supports the bulk RNA-seq findings that the Epi-iBMECs generated by these protocols (1–3) and used in subsequent studies (1, 2, 4–12) correspond to a homogenous epithelial cell population, rather than bona fide brain microvascular ECs capable of forming a BBB. It is of note that our single-cell RNA sequencing dataset includes both in vivo and in vitro brain EC samples along with our other endothelial controls bolstering the conclusion that though Epi-iBMECs may possess properties such as the presence of tight junction proteins and an ability to form a high TEER, they cannot be used as an in vitro model of the human BBB for future studies due to their lack of a basic EC transcriptome.

In this study, we also show that the vascular fate of Epi-iBMECs can be rescued through introduction of appropriate EC-specific ETS transcription factors (*ETV2*, *ERG*, and *FLI1*), which instills an endothelial transcriptomic signature as well as canonical EC functions to Epi-iBMECs in vitro. These reprogrammed cells (rECs) show the capacity to be passaged and expand in vitro, while retaining a PECAM1^+^CDH5^+^KDR^+^ EC immunophenotype. Purified rECs are able to respond to inflammatory stimuli (e.g., TNF-α) and permeabilizing agents (e.g., VEGF-A and anti-VE-cadherin antibody) in a manner canonical to vascular ECs. Similarly to HUVECs, rECs are also able to form tubes in an immunocompromised in vivo mouse model, whereas Epi-iBMECs derived from the same hPSCs could not. We note that our strategy of transcription factor reprogramming was pursued in an effort to establish a vascular EC identity in cells that otherwise lacked any phenotypic and functional aspects of bona fide ECs. Our method illustrates that only upon induction of *ETV2*, *FLI1*, and *ERG* can Epi-iBMECs adopt an EC identity. Though more work needs to be done to produce a reliable brain-specific EC, we suggest that this could be a crucial step toward de novo generation of true BBB-forming brain ECs suitable for in vitro modeling of physiological and pharmaceutical studies which remains an issue with the current Epi-iBMECs.

This work indicates that transcription factor-mediated differentiation may be explored in an effort to generate vascular ECs directly from hPSCs rather than the ectodermal-to-mesodermal reprogramming method described above. Several methods for the generation of ECs directly from hPSCs with varying degrees of vascular organotypicity or phenotypes have been reported using growth factors such as Activin A, BMP4, bFGF, and VEGF-A along with TGBF-β inhibition at various stages of differentiation (27, 54–56). Overexpression of transcription factors such as *SOX18*, *TAL1*, *SOX7*, and *ETS2* enhances BBB properties in generic ECs, increasing barrier resistance and tight junction protein expression while decreasing paracellular transport (57). These studies set forth the evidence that transcription factor overexpression could be used to directly generate ECs from hPSCs, circumventing the need to reprogram another hPSC-derived cell type.

The advent of transcription factor-based cell reprogramming and the understanding that cellular identity is plastic and therefore subject to manipulation has opened new ways to approach and elucidate disease mechanisms, drug development, and cell-based therapeutics for a variety of diseases, including neurological disorders. However, application of rigorous and thorough characterization of stem cell-derived products using the latest available technologies such as single-cell multiomics and metabolomics should be necessary, rather than facultative, for the development of faithful disease models and safe cell-based therapies.

## Methods

### Maintenance of Primary Human Cell Lines.

HUVECs were deidentified and isolated in accordance with Weill Cornell Medicine institutional review board #0804009728R011 as described previously (58). HUVECs were cultured in human endothelial cell media (M199 [Sigma, M4530], 10% fetal bovine serum (FBS) [Omega Scientific, FB07], 50 μg mL^−1^ endothelial mitogen [Alfa Aesar J65416], and 100 μg/mL heparin). Brain microvascular endothelial cells were obtained from Sciencell Research Laboratories (cat. no. 1000) and were maintained following ScienCell guidelines.

### Maintenance of hPSCs and Differentiation to Epi-iBMECs.

IMR90-4 (59–62) and H1 cells were obtained from WiCell, while H6 cells were acquired from the laboratory of Dr. Todd Evans at Weill Cornell Medicine.

These protocols were precisely followed as described previously (1–3). All reagents were procured based on these protocols, and each step and duration for differentiation was followed rigorously. IMR90-4 and H6 iPSCs and H1 hESCs were maintained between passages 20 to 42 on Matrigel (BD Biosciences, 354277) in mTeSR, TeSR-E8 medium (STEMCELL Technologies, 85850, 05990), or Stem Flex (Gibco, A3349401). For differentiation, cells were passaged onto Matrigel in mTeSR1 or StemFlex medium and allowed to expand for 3 d. Cultures were then switched to unconditioned medium (UM) lacking bFGF for 6 d. EC medium consisting of human endothelial serum-free medium (hESFM; Gibco, 11111044) supplemented with 20 ng/mL bFGF (Peprotech, 100-18B), 1% platelet-poor plasma-derived bovine serum (Alfa Aesar, J64483AE), and 10 μM all-trans retinoic acid (Sigma, R2625) was then added for an additional 2 d. Cells were then dissociated with Accutase (Invitrogen, 004555-56) and plated onto six-well polystyrene plates or 1.12 cm^2^ Transwell-Clear permeable inserts (0.4-mm pore size) in EC media. Culture plates were coated with a mixture of Collagen IV (400 mg/mL; Sigma, C6745) and Fibronectin (100 mg/mL; Sigma, F1141) in H_2_O for at least 30 min at 37 °C, whereas inserts were incubated for a minimum of 4 h at 37 °C. The resulting purified hPSC-derived Epi-iBMECs were then grown in EC medium for 24 h, after which RA was removed and cells were cultured until the indicated experimental time points.

### hPSC Differentiation to iECs.

hPSCs were maintained exactly as described above and differentiation protocol was performed as described previously (27). Embryoid bodies were generated and cultured in base hPSC medium consisting of StemPro-34 (Gibco, 10639011), nonessential amino acids (Gibco, 11140050), glutamine (Gibco, 35050061), penicillin/streptomycin/amphotericin B (Gibco, 15240096), and β-mercaptoethanol (Millipore Sigma, M3148) for 24 h before the start of differentiation and the protocol was carried out as previously described. Medium was changed every 2 d for the duration of the differentiation. Addition of 20 ng/mL BMP-4 (R&D Systems, 314-BP-05/C) on day 0 (removed on day 7); on day 1, medium was supplemented with 10 ng/mL activinA (STEMCELL Technologies, 78001; removed at day 4); on day 2, medium was further supplemented with 8 ng/mL FGF-2 (Peprotech, 10018B; remained for the duration of culture); on day 4, embryoid bodies were transferred to adherent conditions on Matrigel-coated plates and medium was supplemented with 25 ng/mL VEGF-A (Peprotech, 100-20; remained for the duration of culture) to specify vascular progenitors; on day 7, SB431542 (R&D Systems, 1614; TGF-β signaling inhibitor) was added at 10 μM concentration and remained for the indicated duration to expand terminally differentiated endothelial cells (iECs).

### Reprogramming Epi-iBMECs into rECs.

IMR90-4 hPSCs were differentiated according to the Epi-iBMEC protocol as described above. Following the 6 d of differentiation in UM, cells were transduced via lentiviral vectors for ETS transcription factors FLI1, ERG, and ETV2 and cultured in EC medium, exactly as mentioned above. No further changes were made to the protocol because the cells were terminally cultured.

### Bulk RNA Sequencing.

Total RNA, greater than 100 ng, from cultured cells was isolated in TRIzol L and purified using Qiagen RNeasy Mini Kit per manufacturer’s protocols. An Agilent Technologies 2100 Bioanalyzer was used to assess the RNA quality. RNA libraries were prepared and multiplexed using Illumina TruSeq RNA Library Preparation Kit v2 (nonstranded and poly-A selection), and 10 nM of cDNA was used as the input for high-throughput sequencing via Illumina’s HiSEq. 2500 platform, producing 50-bp paired end reads. Previously published RNA-seq data for iBMECs and various EC and epithelial cell types were downloaded from the Gene Expression Omnibus (GEO accession numbers: GSE40291, GSE57662, GSE131039, GSE137786, GSE82207, GSE129290, GSE122588, GSE97100, GSE97324, GSE73721, GSE97575, GSE108012, GSE151976, GSE157852, and GSE126449) which were then processed in the same way as for the bulk RNA-seq libraries prepared for the experiment.

Sample files were checked for sequence quality (FastQC v0.11.5) and processed using the Digital Expression Explorer 2 (DEE2) (63) workflow. Adapter trimming was performed with Skewer (v0.2.2) (64). Further quality control was done with Minion, a part of the Kraken package (65). The resultant filtered reads were mapped to human reference genome GRCh38 using STAR aligner (66) and gene-wise expression counts generated using the “-quantMode GeneCounts” parameter. After further filtering and quality control, R package edgeR (67) was used to calculate trimmed mean of M-values (TMM) normalization factors. Fragments per kilobase of transcript per million mapped reads (FPKM) and Log2 counts per million (cpm) matrices were quantified using these factors to normalize for library size. Principal component analysis and Pearson correlation values were quantified with the resulting Log2 cpm values. Gene enrichment analysis was run using WebGestalt (68). Heatmaps were formulated with distance between rows and columns calculated by Euclidean distance.

### Single-Cell RNA-Seq Digital Droplet Sequencing (ddSeq).

A single-cell suspension was loaded into the Bio-Rad ddSeq Single-Cell Isolator on which cells were isolated, lysed, and barcoded in droplets. Droplets were then disrupted, and cDNA was pooled for second strand synthesis. Libraries were generated with direct tagmentation followed by 3′ enrichment and sample indexing using Illumina Nextera library prep kit. Pooled libraries were sequenced on the Illumina NextSeq500 sequencer. Sequencing data were primarily analyzed using the SureCell RNA Single-Cell App in Illumina BaseSpace Sequence Hub. Single-cell RNA-sequencing data using both 10× Genomics Chromium Single-Cell 3′ v3 RNA sequencing and SMART-Seq v4 libraries from the Allen Brain Map that identified human brain endothelial cells were also downloaded from https://portal.brain-map.org/. The 10× Genomics Chromium Single-Cell 3′ v3 RNA-sequencing data from the human choroid plexus were downloaded from GEO accession number GSE150903.

All single-cell analyses were performed using the Seurat package in R (version 3.2.2). For the ddSeq libraries prepared for this publication, UMI count files that had been knee filtered were downloaded from the Illumina BaseSpace Sequence Hub. After initial quality control, cells that were included in the analysis were required to have a minimum of 900 genes expressed and a maximum 5,500 genes expressed, in addition to a minimum 1,100 UMIs resulting in a total of 29,235 cells passing quality filters across the 27 samples as seen in *SI Appendix*, Fig. S7*B*. After downloading processed count matrices and metadata from the Allen Brain Map single-cell RNA-seq, cells with subclass_label “endothelial” and that had expression of Claudin-5 (*CLDN5)* > 0 were selected for further analysis in Seurat resulting in 55 cells from the 10× chromium libraries and 64 cells from SMART-Seq v4 libraries. Day 46 choroid plexus 10× single-cell RNA-seq data were downloaded from GSE150903 and after initial quality control, cells that were included in the analysis were required to have a minimum of 600 genes expressed and a maximum of 9,000 genes expressed, in addition to maximal percentage mitochondrial reads of 30%. This resulted in a total of 3,585 cells passing quality filters. Total cells of the combined sample set were 32,939.

Following best practices in the Seurat package suggestions for sample integration and batch correction the samples were split into four batches (ddSeq, Allen 10×, Allen SMART-seq, and CP D46 10×) and SCTransform was applied to the cells in each batch for normalization. The four batches were then integrated using the Seurat version 3 SCTransform integration and label transfer workflow (69) with nfeatures set at 10,000. Principal component analysis was subsequently performed on the integrated sample and after reviewing principal component heatmaps and jackstraw plots UMAP visualization was performed using the top 40 components. Differential gene expression for gene marker discovery across the clusters and samples was performed using the Wilcoxon rank sum test as used in the Seurat package.

### Endothelial Inflammatory Response Assay.

HUVECs as well as IMR90-4 derived Epi-iBMECs and rECs were cultured as a confluent monolayer on plastic tissue culture plates. A total of 100 ng mL^−1^ of tumor necrosis factor alpha (TNFa) was added to culture media and allowed to stimulate cells for 3 h. Cells were then washed, fixed, and assayed for E-Selectin (CD62E) by confocal microscopy and flow cytometry.

### Immunofluorescence.

#### Fixed samples.

Cells were fixed with 4% paraformaldehyde for 15 min prior to staining. To prevent nonspecific binding of the primary antibody, samples were blocked with 5% horse serum in phosphate-buffered saline (PBS) solution for 60 min. Cells were washed three times in PBS and dilute primary antibody in 5% horse serum was added according to the manufacturer’s recommended concentration and left overnight at 4 °C. If the protein was intracellular, permeabilization using 0.2% Triton X-100 was necessary during block and staining with primary antibody. After staining with primary antibody, cells were incubated with secondary antibody in 5% horse serum for 1 h at room temperature on a shaker. Following a thorough wash to remove unbound secondary antibody, DAPI was used at 1 µg/mL to stain nuclei.

#### Live samples.

Human IgG antibody was used at 1:50 in PBS with 0.5% bovine serum albumin (BSA) and 2 mM ethylenediaminetetraacetic acid (EDTA) to block F_c_ receptors prior to staining. The blocked cells were stained for 30 min at 4 °C with fluorochrome-conjugated antibodies according to the manufacturer’s recommendations. Stained cells were washed in PBS to prevent saturation of the fluorescent dye during imaging. All imaging was performed using a Zeiss 710 META confocal microscope.

### Flow Cytometry.

Before staining, F_c_ receptors were blocked with human IgG antibody at 1:50 (Biolegend) in PBS (pH 7.2) containing 0.5% BSA (fraction V) and 2 mM EDTA for 10 min at 4 °C. For cell surface staining, samples were stained for 30 min at 4 °C with fluorochrome-conjugated antibodies according to the manufacturer’s recommendation. Stained cells were washed in blocking buffer and fixed in 1% paraformaldehyde (PFA) in PBS (pH 7.2) with 2 mM EDTA for flow analysis or resuspended in PBS (pH 7.2) with 2 mM EDTA and 1 μg/mL DAPI (Biolegend) for sorting. Samples were analyzed using a FACS ARIA II SORP (BD Biosciences). Data were collected and analyzed using FACs DIVA 8.0.1 software (BD Biosciences). All gating was determined using unstained controls and fluorescence minus one strategy.

### In Vivo Tubulogenesis Assay.

H1, H6, and IMR90-4 derived Epi-iBMECs at day 16 of differentiation, rECs, and primary HUVECs were resuspended in Matrigel (Corning, 254277) at 500,000 cells/300 μL and injected s.c. into immunocompromised mice (NSG-SGM3; Jax, 013062). Mice were also injected with an empty Matrigel control. After 5 d, mice were killed following institutional guidelines and solidified Matrigel plugs were excised and fixed using 4% paraformaldehyde (Alfa Aesar, AA43368-9M). Plugs were cryosectioned, stained using CD144 (BV9, Biolegend), Agglutinin 1 (UEA1, Vector Labs), and EPCAM (9C4, BioLegend). Slides were fixed using Fluoroshield with DAPI (Sigma-Aldrich, F6057) and imaging was performed using a Zeiss 710 META confocal microscope. Quantification analysis of images was conducted using Angiotool (43) set up according to https://ccrod.cancer.gov/confluence/display/ROB2/Quick+Guide. Settings were calibrated for a vessel thickness of 12, thresholds of 20 to 255, small particle filter of 1,000, and fill holes control set to 1,500.

### Virus Production.

Human ETV2, FLI1, and ERG lentiviral particles were generated and titered as described in Ginsberg et al. (70), with the following modifications. The 293T Lenti-X cells (passages 8 to 10; subconfluent) were used to produce lentiviral particles using the Lenti-X packaging single-shot system, following the manufacturer’s protocol. Filter was through a 0.45-μm PES filter attached to a 30-mL syringe and Lenti-358 × concentrator was used following the manufacturer’s protocol. The concentrate was resuspended from a 100-mm plate of 293T cells in 100 μL of lentivirus storage buffer and stored at −80 °C (for up to 1 y).

### Trans-Endothelial Electrical Resistance Measurement.

Cells were plated on poly-D-lysine and Fibronectin and Collagen IV-coated gold electroarray 96-well plates (Applied Biophysics) at day 8 of their initial differentiation. Cells were grown in the presence of media and growth factors described above until they reached maximum resistance for 6 d. The resistance was recorded every 3 h for 6 d using the ECIS Z-θ system (Applied Biophysics). Resistance curves were generated using GraphPad Prism software.

### Animal Experiments.

Mice (12- to 15-wk-old immunodeficient), specifically NSG-SGM3, Jax, 013062, were used in this study. All animal experiments were performed under the approval of the Weill Cornell Medicine Institutional Animal Care and Use Committee (#2009–0061).

### Permeability Assay.

HUVECs, IMR90-4 derived Epi-iBMECs, and rECs were cultured as a monolayer onto Transwell plates with 0.4-μm pore sized inserts (Corning). Cells were grown till confluent at which time 50 ng/mL anti-VE-cadherin antibody (BV9) or 100 ng/mL VEGF-A was added to the cell culture media. Seventy kilodaltons of rhodamine-labeled dextran (Invitrogen) was then pipetted in each well and allowed to incubate for 6 h. Media passed through the Transwell filters were then collected and analyzed on a spectrophotometer (Bio-Rad) measuring the amount of dextran allowed to pass through the cell monolayers at each condition.

## Supplementary Material

Supplementary File

Supplementary File

Supplementary File

## Data Availability

RNA-sequencing data have been deposited in GEO (GSE138025).
