# Supplementary material for: Pluripotent stem cell-derived epithelium misidentified as brain microvascular endothelium requires ETS factors to acquire vascular fate
Source: Proc Natl Acad Sci U S A. 2021 Feb 4;118(8):e2016950118. doi: 10.1073/pnas.2016950118 (PMC7923590; doi:10.1073/pnas.2016950118)

Supplementary Information

Pluripotent stem cell-derived epithelium misidentified as brain microvascular endothelium require ETS-factors to acquire vascular fate.

Tyler M. Lu[1,2], Sean Houghton[1], Tarig Magdeldin[3], José Gabriel Barcia Durán[1], Andrew P. Minotti[4,5], Amanda Snead[6,7], Andrew Sproul[6,7], Duc-Huy T. Nguyen[1], Jenny Xiang[8], Howard A. Fine[3], Zev Rosenwaks[2], Lorenz Studer[5], Shahin Rafii[1], Dritan Agalliu[6,9], David Redmond[1]* and Raphaël Lis[1,2]*

* To whom the correspondance may be addressed.

Raphaël Lis ral2020@med.cornell.edu

David Redmond dar2042@med.cornell.edu

Supplemental Figure legends

Figure S1. Multidimensional Scaling Analysis of bulk RNA sequencing of hPSC-derived cells and their functional characterization

- **A.** Multidimensional Scaling (MDS) plot approximating relative relationship of 109 distinct cell samples across 22 library preparations from previously published and newly generated bulk RNA sequencing data.
- **B.** Scree plot showing the contribution of PC1-6 to the explained variances observed across gene expression profiles for all samples.
- **C.** Immunofluorescence for ZO-1 (green), OCCLUDIN (red) and DAPI (blue) in IMR90-4 derived Epi-iBMECs (day 14) using the published antibodies. These antibodies recognize proteins at cell-cell junctions. Scale bars = 40µm.
- **D.** Measurement of Trans-Endothelial Electrical Resistance (TEER) and Capacitance of IMR90-4 derived Epi-iBMECs over time using an ECIS instrument. The cells were plated on an ECIS plate coated with Fibronectin and Collagen IV substrate on day 8 of the neuro-endothelial differentiation (See Fig. 1a) and TEER was measured for 6 days. Epi-iBMECs form a high TEER by day 14 consistent with prior studies.
- **E.** Immunofluorescence for ZO-1 (red), OCCLUDIN (red) and DAPI (blue) in IMR90-4 derived rECs. Scale bars = 50µm
- **F.** Permeability assay comparing Dextran (70kD) permeability through a monolayer of HUVEC (red), Epi-iBMEC (purple), and rEC (green) when treated with anti-VE-cad antibody or VEGF-A.
- **G.** Measurement of Trans-Endothelial Electrical Resistance (TEER) Epi-iBMECs and purified rECs over time using an ECIS instrument. Measurements were acquired starting day 8 of Epi-iBMEC differentiation.

Figure S2. Gene set enrichment analysis of PC1 And PC2 loading genes.

- **A.** Gene set enrichment analysis of biological processes using all PC1 loading genes derived from Fig. 1c highlighting major causes of divergence across all samples along PC1 of the principal component analysis.
- **B.** Gene set enrichment analysis of biological processes using all PC2 loading genes derived from Fig. 1c highlighting major causes of divergence across all samples along PC2 of the principal component analysis.

Figure S3. Principal Component Analysis and Multidimensional Scaling Analysis of hPSC-derived cells and their gene expression characterization.

- **A.** Principal Component Analysis plot approximating relative relationship of 113 distinct cell samples across 23 library preparations from previously published and newly generated bulk RNA sequencing data. rECs highlighted to group with endothelial cells.
- **B.** Multidimensional Scaling (MDS) plot approximating relative relationship of 113 distinct cell samples across 13 library preparations from previously published and newly generated bulk RNA sequencing data. rECs highlighted to group with endothelial cells.
- **C.** Violin plots of key endothelial cell genes acquired from top loading genes of PC1 of the bulk RNA expression profile analysis demonstrating significant difference in expression between rECs and both Epi-iBMEC as well as epithelial cell populations. (significance indicates p-value<0.05)
- **D.** Violin plots of key epithelial cell genes acquired from top loading genes of PC1 of the bulk RNA expression profile analysis demonstrating significant difference in expression between rECs and both Epi-iBMEC as well as epithelial cell populations. (significance indicates p-value<0.05)

Figure S4. Violin plots of brain endothelial cell transporter genes.

Violin plots representing expression levels of brain endothelial cell transporter genes in the bulk RNA sequencing analysis of all cell samples from Fig. 1c as well as rECs. Transporter genes were selected via differential expression analysis of the Allen Institute Brain Atlas.

Figure S5. Violin plots of the glucose transporter genes.

Violin plots representing expression levels of glucose transporters in the bulk RNA sequencing analysis of all cell samples from Fig. 1c as well as rECs.

Figure S6. Violin plots of claudin genes.

Violin plots representing expression levels of Claudin genes in the bulk RNA sequencing analysis all cell samples from Fig. 1c as well as rECs.

Figure S7. Analysis of expression of endothelial proteins in rEC.

A. Confocal microscopy for EpCAM (purple), PECAM1 (green), and CDH5 (red) in rECs at days 12 and 26 (14 days after FACS purification). White arrows show regions of junctional expression of CDH5 that is restored in rECs. n=5 biological replicates. Scale bars = 200μm.

B. Violin plot describing the total number of cells per sample that passed quality control and were used in the study. The number of genes detected per cell is reported on the x axis.

Figure S8. Principle component analysis of single cell RNA sequencing data from iPSC-derived Epi-iBMECs and rECs.

- **A.** Principal component analysis of single cell RNA sequencing data displaying 32,939 cells from 30 distinct cell samples. All endothelial cells are shown to diverge from Epi-iBMEC and choroid plexus organoid cells across PC1.
- **B.** Gene set enrichment analysis of biological processes separating all positive and negative PC1 loading genes from Fig. S7A highlighting the major causes for divergence across PC1.
- **C.** Venn diagrams comparing all positive and negative PC1 genes from both the bulk RNA sequencing analysis (Fig. 1C) and single cell sequencing analysis (Fig. S7A). Approximately 80% of all genes influencing sample divergence in either positive or negative directions across PC1 of the single-cell sequencing principal component analysis intersect with loading genes from the bulk RNA sequencing analysis.

Figure S9. Heatmap comparison in expression levels of endothelial cell specific genes.

- **A.** Heatmap emphasizing differences in expression levels of endothelial cell specific genes as defined by the Allen Brain Institute in all cell samples from Fig. 2B.
- **B.** Heatmap emphasizing differences in expression levels of brain microvascular markers as defined previously by Song et al. (2020) in all cell samples from Fig. 2B.

Supplemental Dataset legends

Dataset S1. FPKM matrix of bulk RNA-seq data showing differential gene expression across all samples used.

Dataset S2. Detailed gene and sample list corresponding to various figures as well as antibody lists.

Figure S1

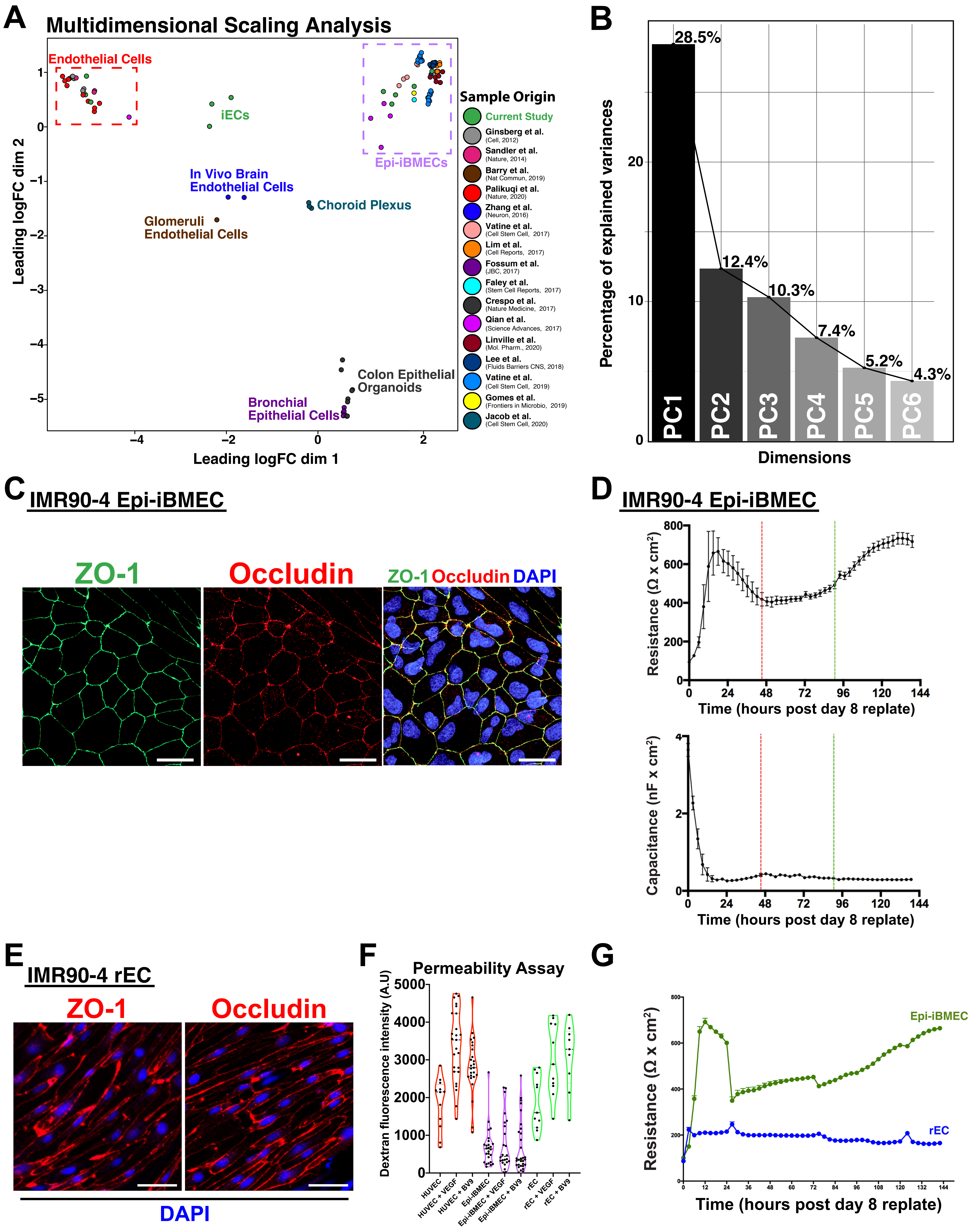
A
Multidimensional Scaling Analysis
Endothelial Cells
iECs
Epi-iBMECs
In Vivo Brain
Endothelial Cells
Choroid Plexus
Glomeruli
Endothelial Cells
Colon Epithelial
Organoids
Bronchial
Epithelial Cells
Leading logFC dim 1
Leading logFC dim 2
Sample Origin
Current Study
Ginsberg et al. (Cell, 2012)
Sandler et al. (Nature, 2014)
Barry et al. (Nat Commun, 2019)
Palikuqi et al. (Nature, 2020)
Zhang et al. (Neuron, 2016)
Vatine et al. (Cell Stem Cell, 2017)
Lim et al. (Cell Reports, 2017)
Fossum et al. (JBC, 2017)
Faley et al. (Stem Cell Reports, 2017)
Crespo et al. (Nature Medicine, 2017)
Qian et al. (Science Advances, 2017)
Linville et al. (Mol. Pharm., 2020)
Lee et al. (Fluids Barriers CNS, 2018)
Vatine et al. (Cell Stem Cell, 2019)
Gomes et al. (Frontiers in Microbio, 2019)
Jacob et al. (Cell Stem Cell, 2020)
B
Percentage of explained variances
Dimensions
28.5%
12.4%
10.3%
7.4%
5.2%
4.3%
PC1
PC2
PC3
PC4
PC5
PC6
C
IMR90-4 Epi-iBMEC
ZO-1
Occludin
ZO-1 Occludin DAPI
D
IMR90-4 Epi-iBMEC
Resistance (Ω x cm²)
Time (hours post day 8 replate)
Capacitance (nF x cm²)
Time (hours post day 8 replate)
E
IMR90-4 rEC
ZO-1
Occludin
DAPI
F
Permeability Assay
Dextran fluorescence intensity (A.U)
HUVEC
HUVEC + VEGF
HUVEC + BV9
Epi-iBMEC
Epi-iBMEC + VEGF
Epi-iBMEC + BV9
rEC
rEC + VEGF
rEC + BV9
G
Resistance (Ω x cm²)
Epi-iBMEC
rEC
Time (hours post day 8 replate)

Figure S2

A

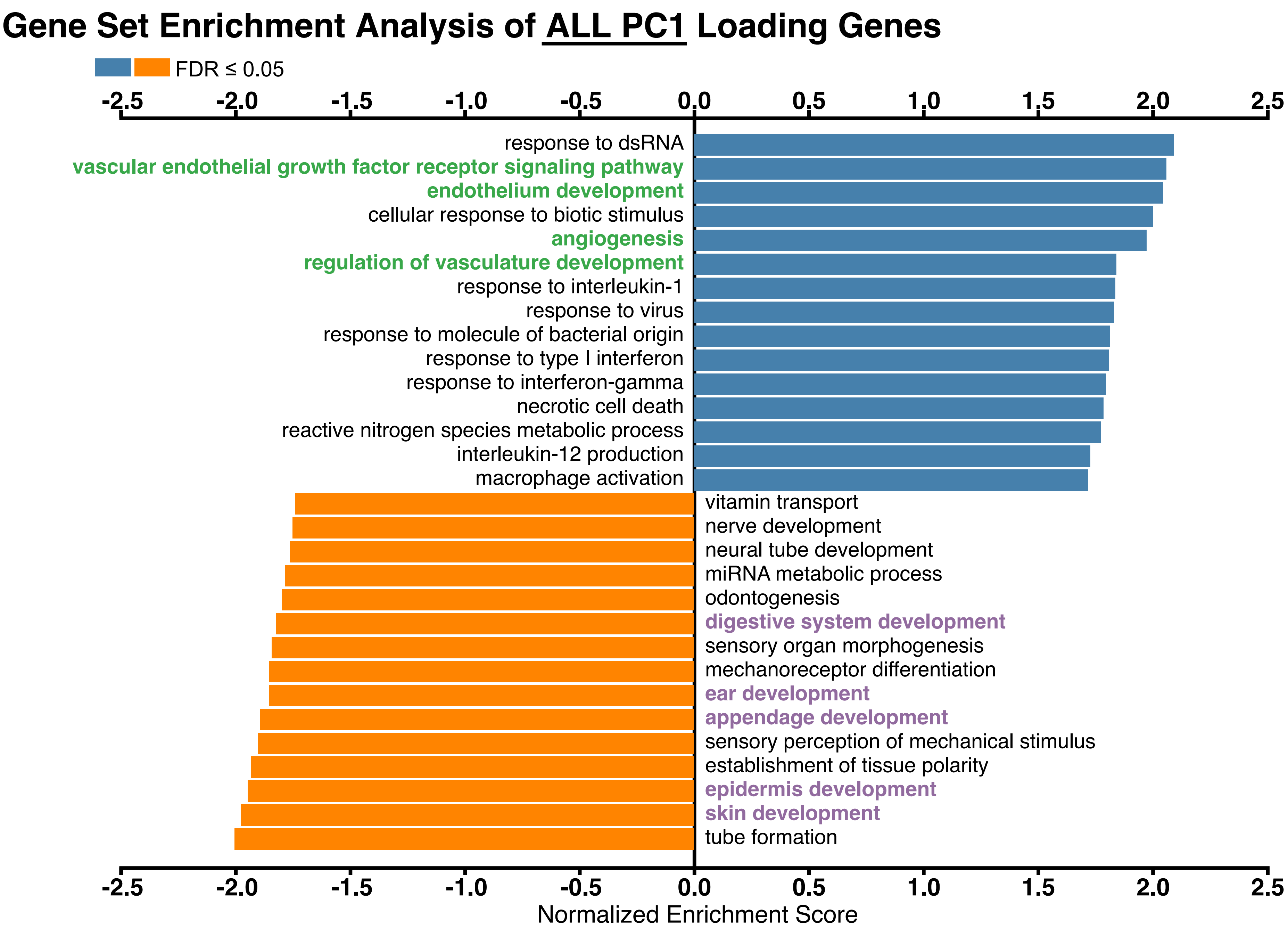

Gene Set Enrichment Analysis of ALL PC1 Loading Genes
FDR ≤ 0.05
-2.5 -2.0 -1.5 -1.0 -0.5 0.0 0.5 1.0 1.5 2.0 2.5
response to dsRNA
vascular endothelial growth factor receptor signaling pathway
endothelium development
cellular response to biotic stimulus
angiogenesis
regulation of vasculature development
response to interleukin-1
response to virus
response to molecule of bacterial origin
response to type I interferon
response to interferon-gamma
necrotic cell death
reactive nitrogen species metabolic process
interleukin-12 production
macrophage activation
vitamin transport
nerve development
neural tube development
miRNA metabolic process
odontogenesis
digestive system development
sensory organ morphogenesis
mechanoreceptor differentiation
ear development
appendage development
sensory perception of mechanical stimulus
establishment of tissue polarity
epidermis development
skin development
tube formation
Normalized Enrichment Score

B

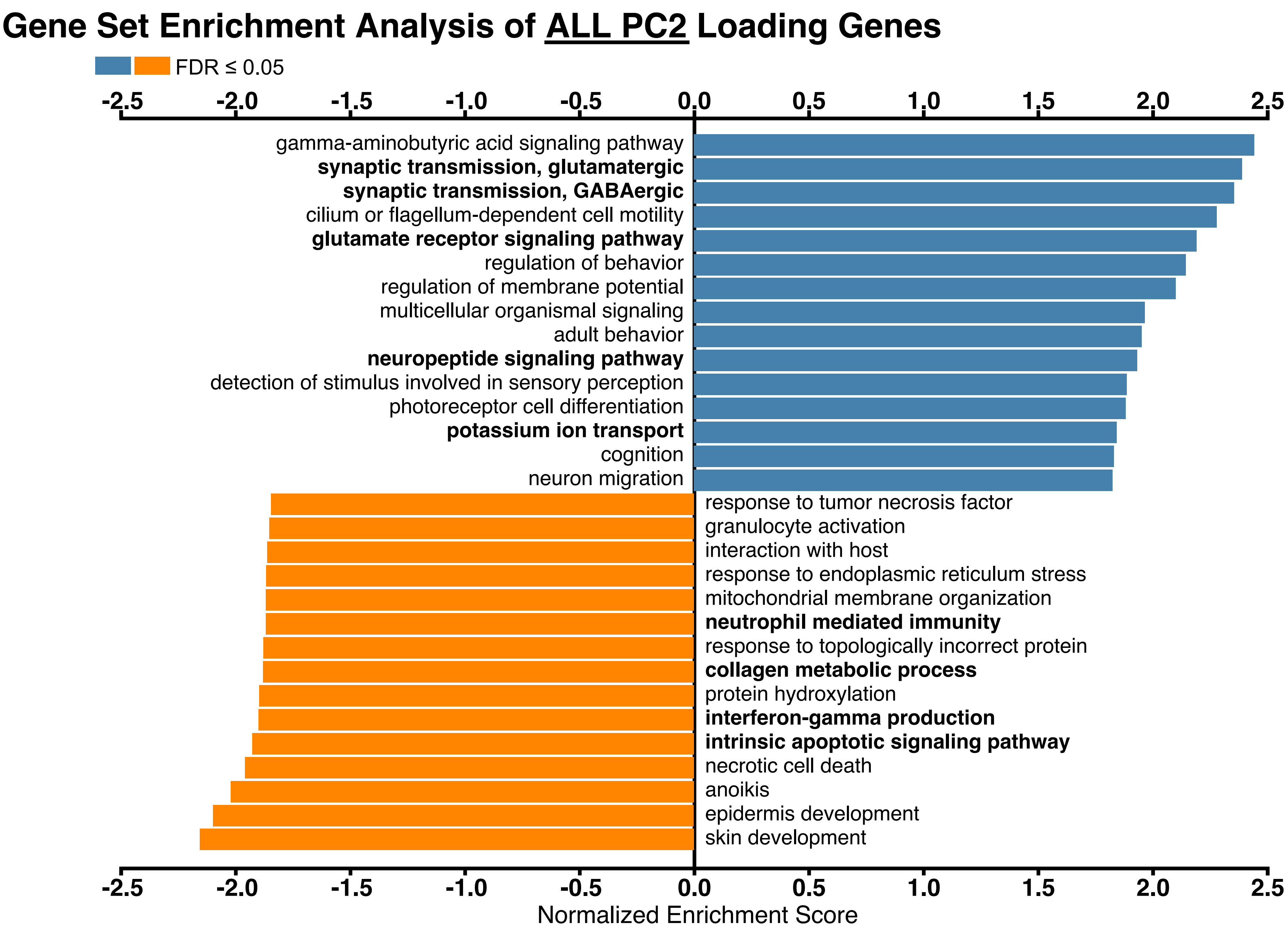

Gene Set Enrichment Analysis of ALL PC2 Loading Genes
FDR ≤ 0.05
-2.5 -2.0 -1.5 -1.0 -0.5 0.0 0.5 1.0 1.5 2.0 2.5
gamma-aminobutyric acid signaling pathway
synaptic transmission, glutamatergic
synaptic transmission, GABAergic
cilium or flagellum-dependent cell motility
glutamate receptor signaling pathway
regulation of behavior
regulation of membrane potential
multicellular organismal signaling
adult behavior
neuropeptide signaling pathway
detection of stimulus involved in sensory perception
photoreceptor cell differentiation
potassium ion transport
cognition
neuron migration
response to tumor necrosis factor
granulocyte activation
interaction with host
response to endoplasmic reticulum stress
mitochondrial membrane organization
neutrophil mediated immunity
response to topologically incorrect protein
collagen metabolic process
protein hydroxylation
interferon-gamma production
intrinsic apoptotic signaling pathway
necrotic cell death
anoikis
epidermis development
skin development
Normalized Enrichment Score

Figure S3

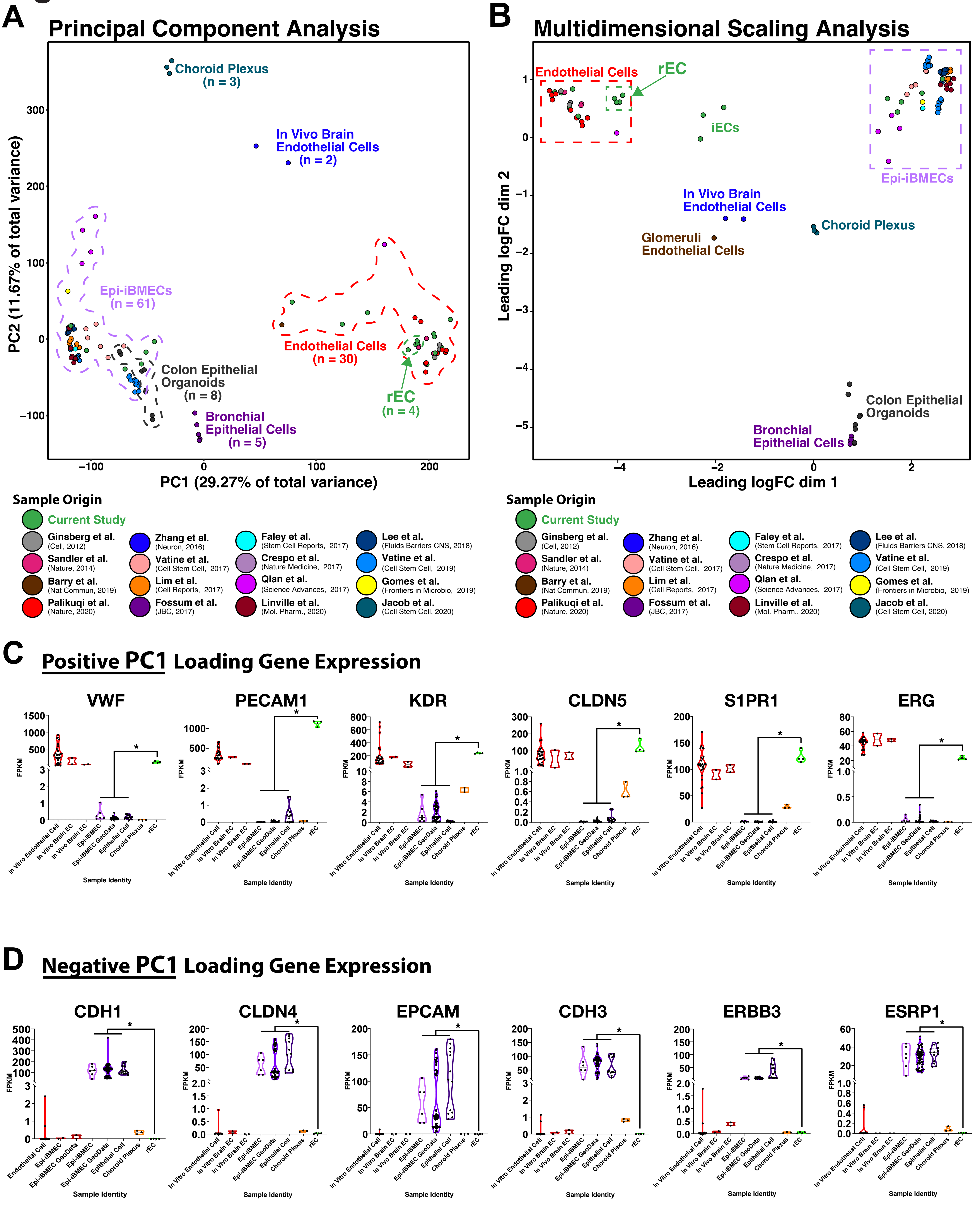
A
Principal Component Analysis
Choroid Plexus (n = 3)
In Vivo Brain Endothelial Cells (n = 2)
Epi-iBMECs (n = 61)
Endothelial Cells (n = 30)
Colon Epithelial Organoids (n = 8)
rEC (n = 4)
Bronchial Epithelial Cells (n = 5)
PC2 (11.67% of total variance)
PC1 (29.27% of total variance)
B
Multidimensional Scaling Analysis
Endothelial Cells
rEC
iECs
Epi-iBMECs
In Vivo Brain Endothelial Cells
Choroid Plexus
Glomeruli Endothelial Cells
Colon Epithelial Organoids
Bronchial Epithelial Cells
Leading logFC dim 2
Leading logFC dim 1
Sample Origin
Current Study
Ginsberg et al. (Cell, 2012)
Sandler et al. (Nature, 2014)
Barry et al. (Nat Commun, 2019)
Palikuqi et al. (Nature, 2020)
Zhang et al. (Neuron, 2016)
Vatine et al. (Cell Stem Cell, 2017)
Lim et al. (Cell Reports, 2017)
Fossum et al. (JBC, 2017)
Faley et al. (Stem Cell Reports, 2017)
Crespo et al. (Nature Medicine, 2017)
Qian et al. (Science Advances, 2017)
Linville et al. (Mol. Pharm., 2020)
Lee et al. (Fluids Barriers CNS, 2018)
Vatine et al. (Cell Stem Cell, 2019)
Gomes et al. (Frontiers in Microbio, 2019)
Jacob et al. (Cell Stem Cell, 2020)
C
Positive PC1 Loading Gene Expression
VWF
PECAM1
KDR
CLDN5
S1PR1
ERG
FPKM
In Vitro Endothelial Cell
In Vitro Brain EC
In Vivo Brain EC
Epi-iBMEC
Epi-iBMEC GeoData
Epithelial Cell
Choroid Plexus
rEC
Sample Identity
D
Negative PC1 Loading Gene Expression
CDH1
CLDN4
EPCAM
CDH3
ERBB3
ESRP1
FPKM
Endothelial Cell
Sample Identity

Figure S4

Allen Institute Brain Endothelial Cell Transporter Expression

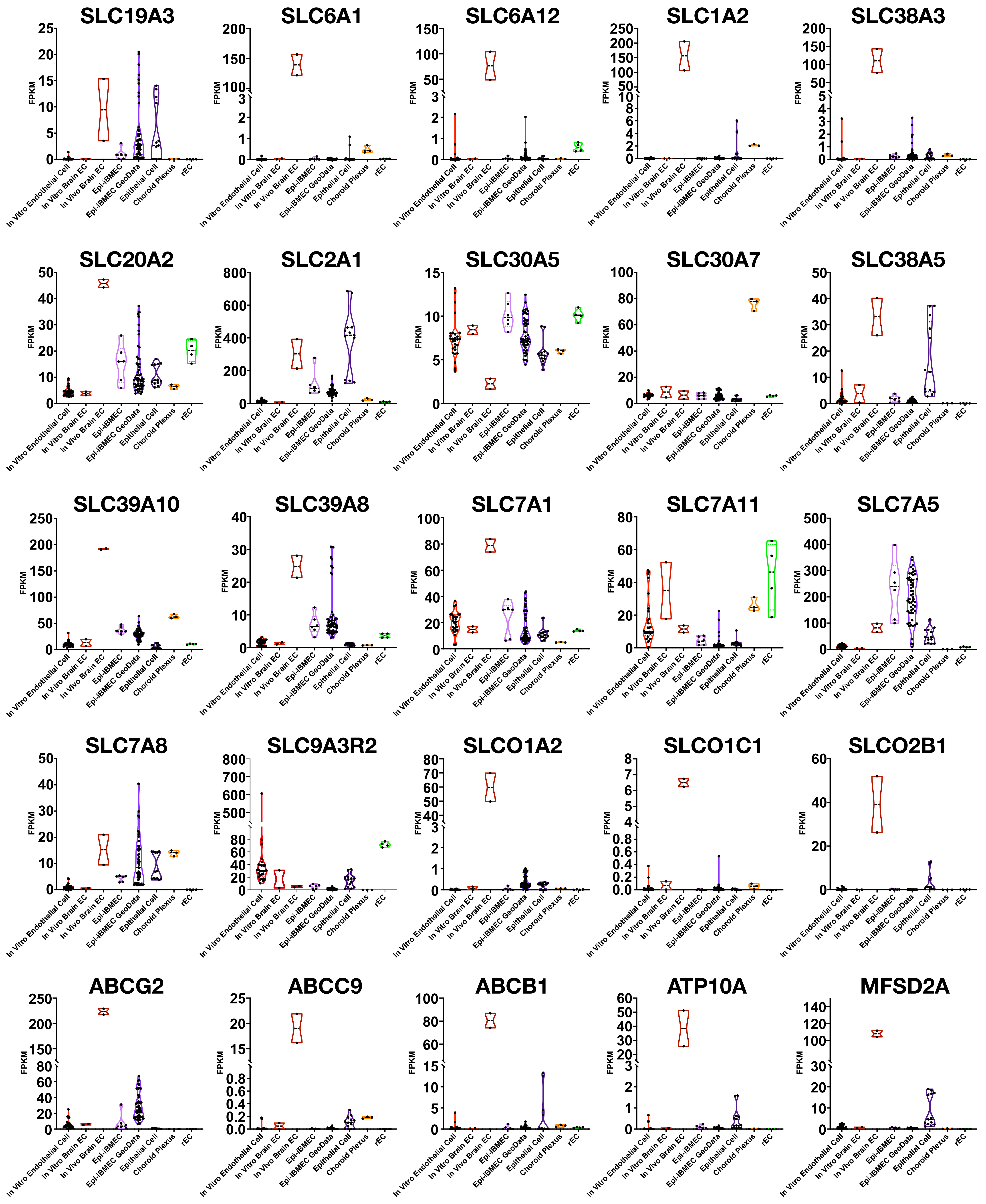

Figure S5

Glucose Transporter Gene Expression

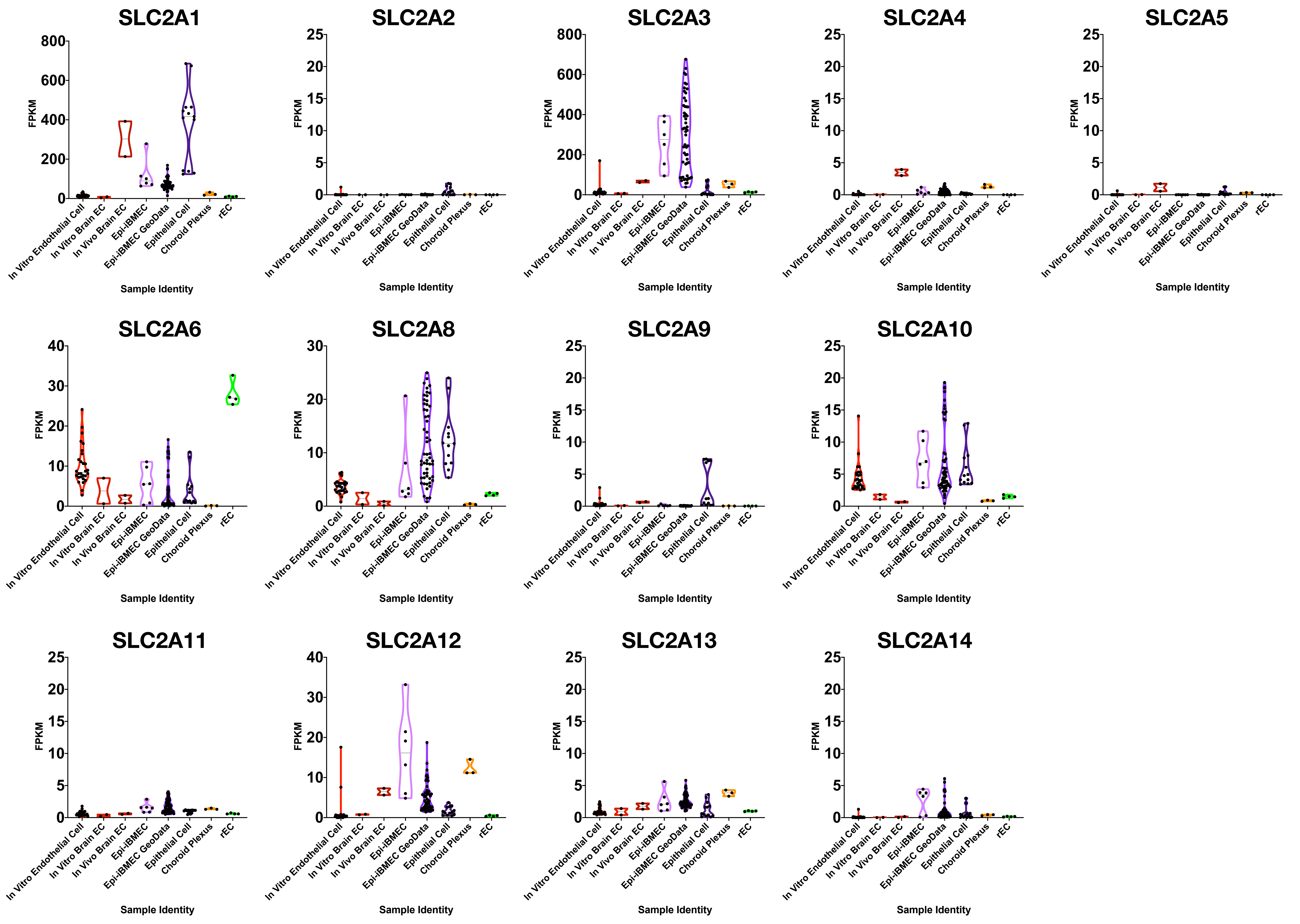

Figure S6

Claudin Gene Expression

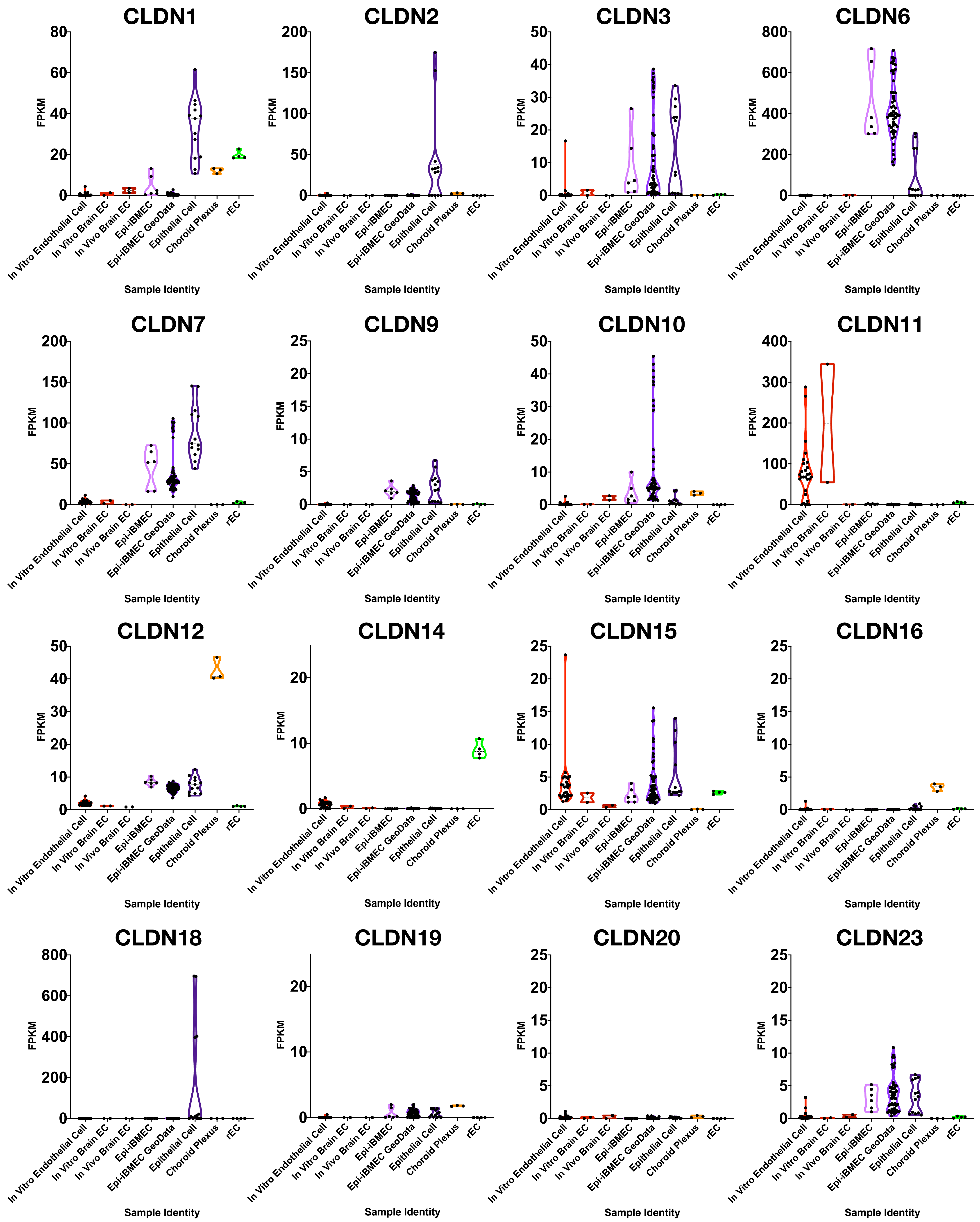

Figure S7

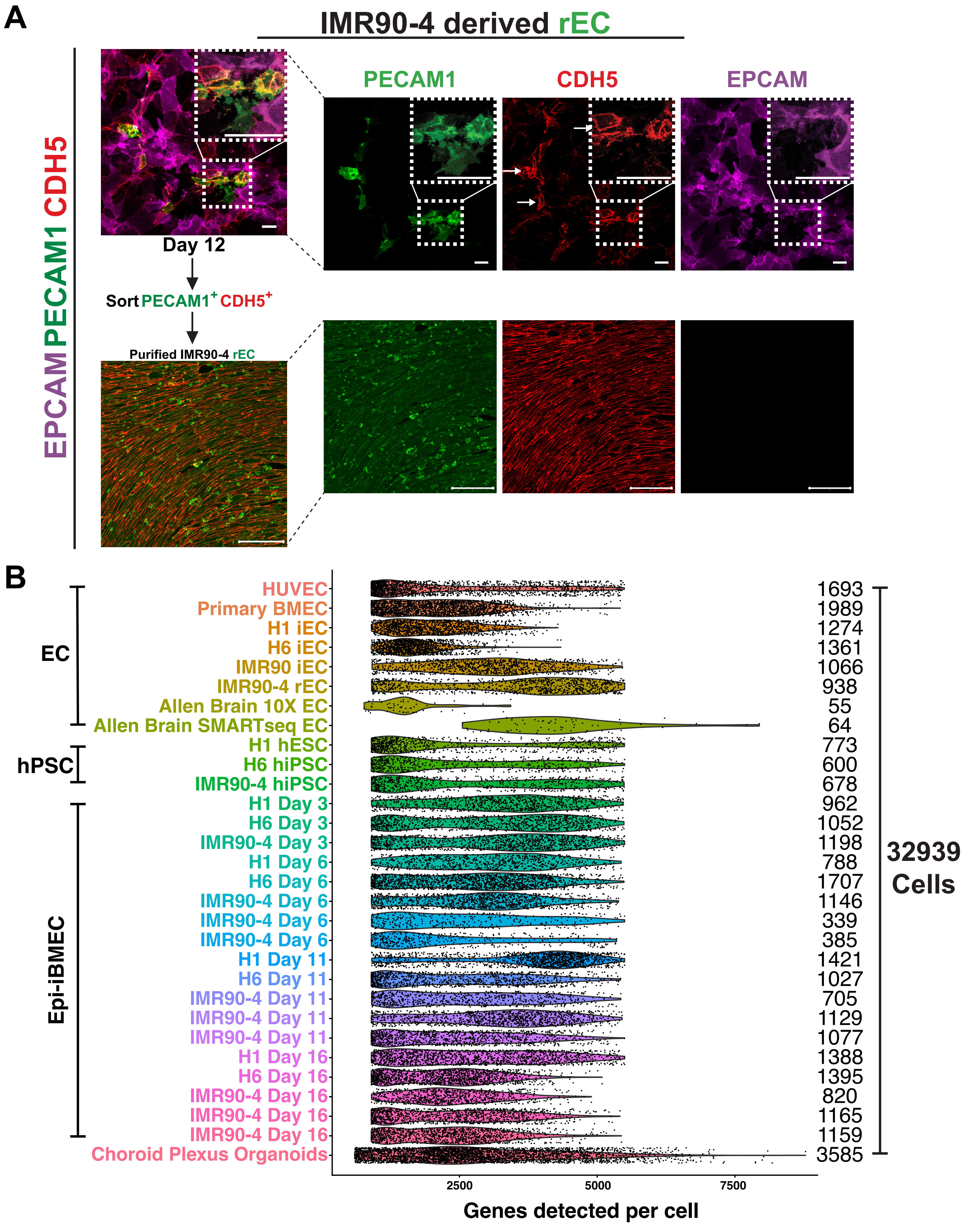
A
IMR90-4 derived rEC
EPCAM PECAM1 CDH5
Day 12
Sort PECAM1+ CDH5+
Purified IMR90-4 rEC
PECAM1
CDH5
EPCAM
B
EC
hPSC
Epi-iBMEC
HUVEC
Primary BMEC
H1 iEC
H6 iEC
IMR90 iEC
IMR90-4 rEC
Allen Brain 10X EC
Allen Brain SMARTseq EC
H1 hESC
H6 hiPSC
IMR90-4 hiPSC
H1 Day 3
H6 Day 3
IMR90-4 Day 3
H1 Day 6
H6 Day 6
IMR90-4 Day 6
IMR90-4 Day 6
IMR90-4 Day 6
H1 Day 11
H6 Day 11
IMR90-4 Day 11
IMR90-4 Day 11
IMR90-4 Day 11
H1 Day 16
H6 Day 16
IMR90-4 Day 16
IMR90-4 Day 16
IMR90-4 Day 16
Choroid Plexus Organoids
1693
1989
1274
1361
1066
938
55
64
773
600
678
962
1052
1198
788
1707
1146
339
385
1421
1027
705
1129
1077
1388
1395
820
1165
1159
3585
32939 Cells
2500
5000
7500
Genes detected per cell

Figure S8

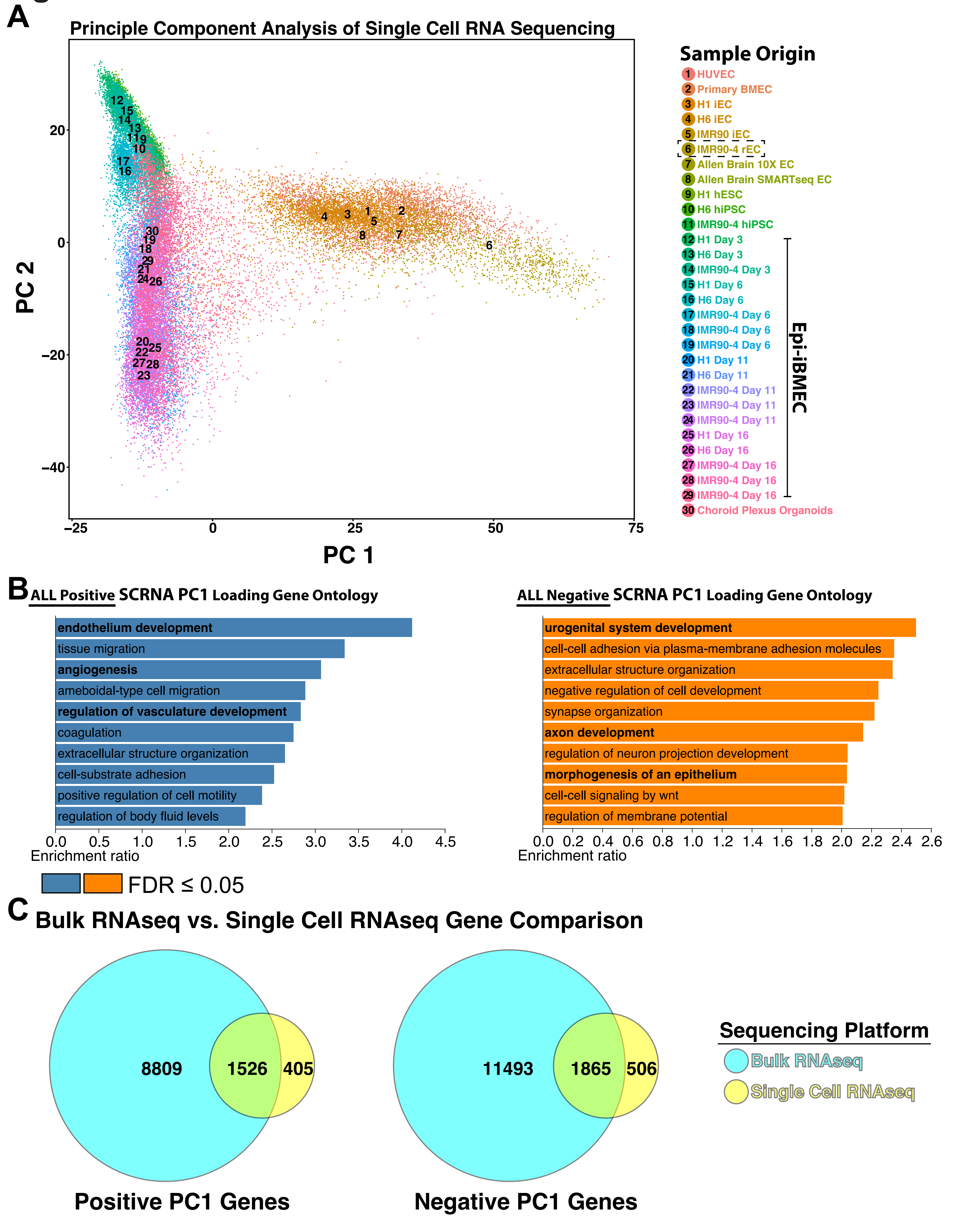
A
Principle Component Analysis of Single Cell RNA Sequencing
PC 2
PC 1
Sample Origin
1 HUVEC
2 Primary BMEC
3 H1 iEC
4 H6 iEC
5 IMR90 iEC
6 IMR90-4 rEC
7 Allen Brain 10X EC
8 Allen Brain SMARTseq EC
9 H1 hESC
10 H6 hiPSC
11 IMR90-4 hiPSC
12 H1 Day 3
13 H6 Day 3
14 IMR90-4 Day 3
15 H1 Day 6
16 H6 Day 6
17 IMR90-4 Day 6
18 IMR90-4 Day 6
19 IMR90-4 Day 6
20 H1 Day 11
21 H6 Day 11
22 IMR90-4 Day 11
23 IMR90-4 Day 11
24 IMR90-4 Day 11
25 H1 Day 16
26 H6 Day 16
27 IMR90-4 Day 16
28 IMR90-4 Day 16
29 IMR90-4 Day 16
30 Choroid Plexus Organoids
Epi-iBMEC
B
ALL Positive SCRNA PC1 Loading Gene Ontology
endothelium development
tissue migration
angiogenesis
ameboidal-type cell migration
regulation of vasculature development
coagulation
extracellular structure organization
cell-substrate adhesion
positive regulation of cell motility
regulation of body fluid levels
Enrichment ratio
ALL Negative SCRNA PC1 Loading Gene Ontology
urogenital system development
cell-cell adhesion via plasma-membrane adhesion molecules
extracellular structure organization
negative regulation of cell development
synapse organization
axon development
regulation of neuron projection development
morphogenesis of an epithelium
cell-cell signaling by wnt
regulation of membrane potential
Enrichment ratio
FDR ≤ 0.05
C
Bulk RNAseq vs. Single Cell RNAseq Gene Comparison
8809
1526
405
11493
1865
506
Positive PC1 Genes
Negative PC1 Genes
Sequencing Platform
Bulk RNAseq
Single Cell RNAseq

Figure S9

A Allen Institute Brain Atlas Endothelial Cell Gene Profile

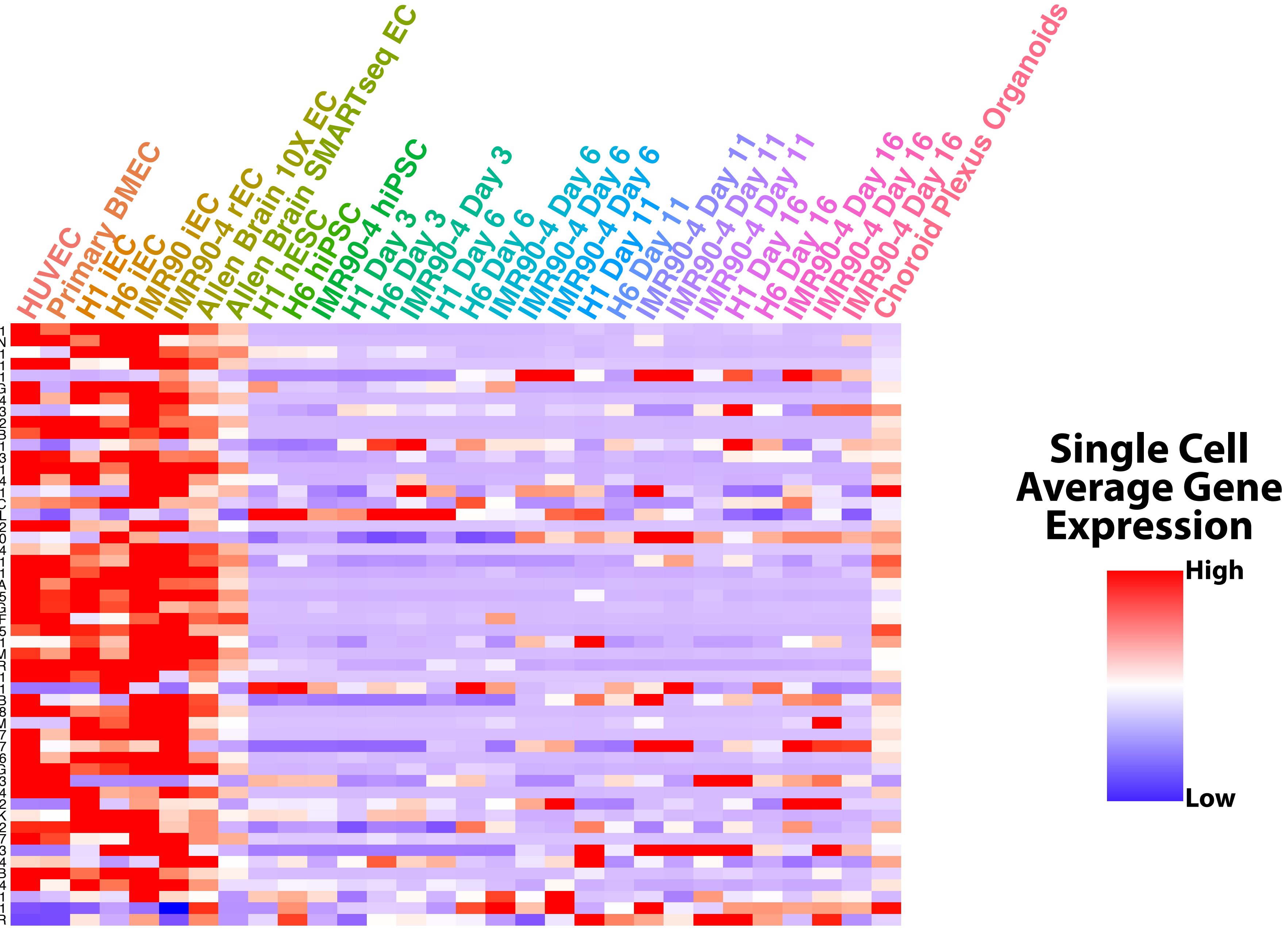

B Song et al. 2020 Brain Microvascular Cell Profile

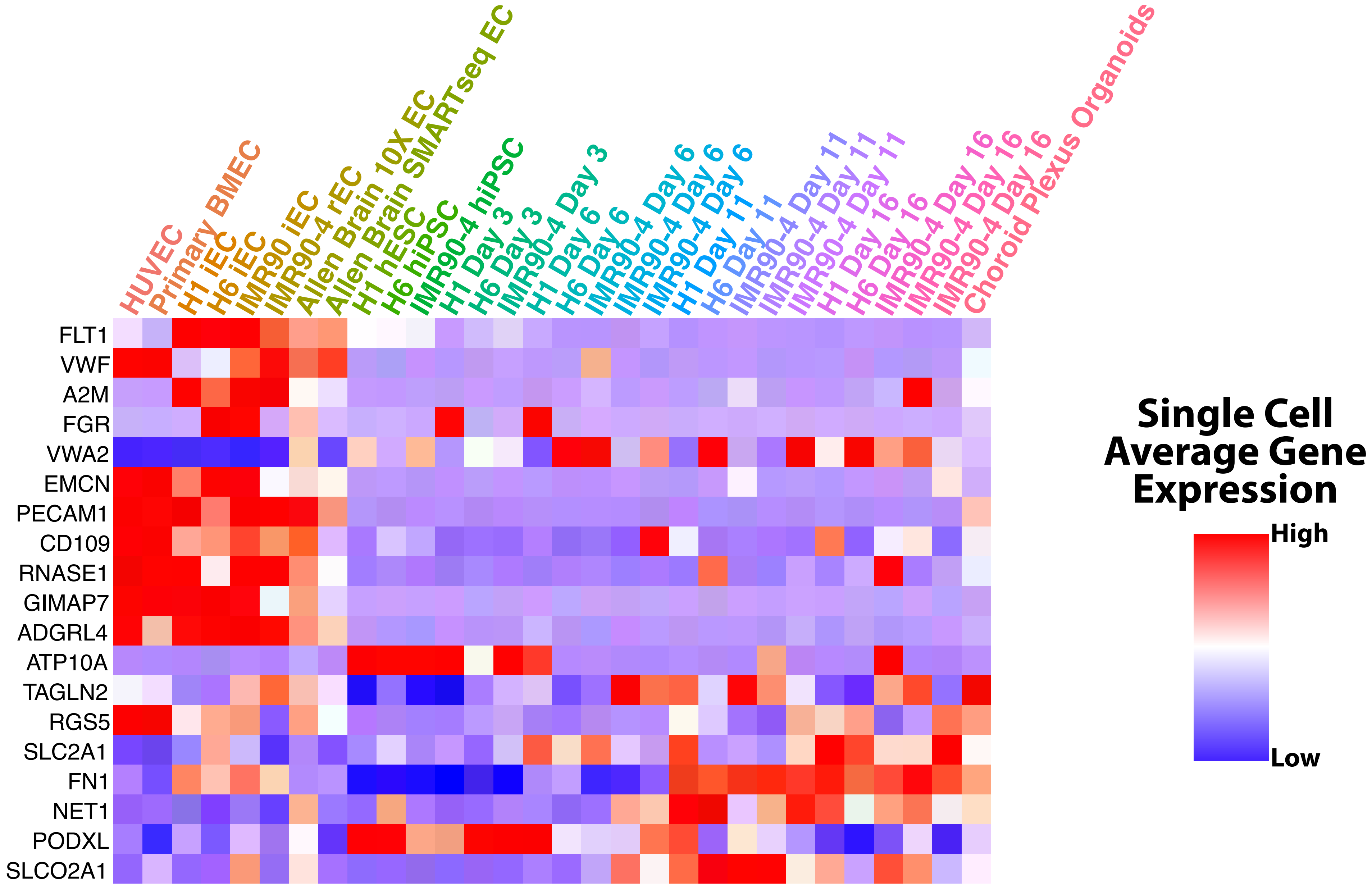

Supplement: Supplementary File [file pnas.2016950118.sapp.pdf]
